# Quantifying the Impact of Patient-Specific Factors and Disease Severity on Clinical Decision Making in Cuff Tear Arthropathy: A Case-Based Survey

**DOI:** 10.1007/s11420-019-09695-x

**Published:** 2019-07-05

**Authors:** Adam P. Schumaier, Yehia H. Bedeir, Joshua S. Dines, Keith Kenter, Lawrence V. Gulotta, David M. Dines, Brian M. Grawe

**Affiliations:** 1 Department of Orthopaedics and Sports Medicine, University of Cincinnati, 231 Albert Sabin Way, Cincinnati, OH USA; 2 Hospital for Special Surgery, New York, NY USA; 3 Western Michigan University Homer Stryker MD School of Medicine, Department of Orthopaedic Surgery, Kalamazoo, MI USA

**Keywords:** rotator cuff, shoulder arthroplasty, reverse shoulder arthroplasty, cuff tear arthropathy, Seebauer, Hamada

## Abstract

**Background:**

Rotator cuff tears are a common cause of disability. Some patients with massive and irreparable tears can develop cuff tear arthropathy (CTA), which makes management more challenging.

**Questions/Purposes:**

We sought to examine how orthopedists determine treatment for patients with CTA. Specifically, we investigated (1) the effect of patient age, symptoms, activity level, range of motion, and radiographic findings on the decision making of shoulder specialists and (2) the observer reliability of the Seebauer and Hamada grading systems.

**Methods:**

Five shoulder surgeons were each sent 108 simulated patient cases. Each simulated patient had a different combination of factors, including patient age, symptoms, activity level, range of motion, and radiographs. Responders graded the radiographs and chose a treatment (non-operative, arthroscopic, hemiarthroplasty, or reverse total shoulder arthroplasty). Spearman’s correlations and *χ*^2^ tests were used to assess the relationship between factors and treatments. Sub-analysis was performed on surgical cases. An intra-class correlation (ICC) was used to assess observer agreement.

**Results:**

The significant Spearman’s correlations were symptoms (0.45), Hamada grade (0.38), patient age (0.34), and Seebauer type (0.29). In sub-analysis of operative cases, the significant correlations were Hamada grade (0.56), patient age (0.51), Seebauer type (0.46), activity level (−0.13). The *χ*^2^ analysis was significant for all factors except activity level. The inter- and intraobserver reliabilities were, respectively, Seebauer type (0.59 and 0.63) and Hamada grade (0.58 and 0.65). Interobserver reliability for patient management was 0.44.

**Conclusion:**

When evaluating CTA, patient symptoms, radiographic grade, and patient age are the factors most strongly associated with the decision making of shoulder specialists. Additionally, the Seebauer and Hamada classifications had similar reliability and clinical utility. However, there was only fair agreement regarding treatment, which indicates that CTA management remains controversial.

**Electronic supplementary material:**

The online version of this article (10.1007/s11420-019-09695-x) contains supplementary material, which is available to authorized users.

**Overview**

Patients with irreparable rotator cuff tears can develop cuff tear arthropathy (CTA), presenting management challenges. The researchers sought to determine what most influences shoulder surgeons in choosing non-operative, arthroscopic, or joint replacement treatment for CTA, as well as the inter- and intraobserver reliability of the Seebauer and Hamada radiographic grading systems. They sent five shoulder surgeons 108 simulated patient cases with varying factors (patient age, symptoms, activity level, range of motion, and radiographs); the surgeons graded the radiographs and chose a treatment for each case. Survey results suggest that patient symptoms, patient age, and radiographic findings have the strongest association with clinical decision making; activity level and range of motion have a weaker association. In cases treated surgically, radiographic grade (disease severity) and patient age were most strongly associated with treatment choice. Both the Seebauer and Hamada classifications showed fair to good inter- and intraobserver reliability.

**Learning Objectives**

Hospital for Special Surgery continuing medical education (CME) activities are intended to improve the quality of patient care and safety. At the conclusion of the activity, the participant should be able to:

• Recognize patient factors that have the greatest effect on clinical decision making in cuff tear arthropathy (CTA) and implement strategies for evaluation and management of CTA in their practice

• Describe the Seebauer and Hamada grading systems and their clinical implications on evaluating CTA

**Target Audience**

This activity is targeted to orthopedic surgeons. Physician assistants, radiologists, residents, fellows, and medical students may also benefit from completing this activity.

**Accreditation**

Hospital for Special Surgery is accredited by the Accreditation Council for Continuing Medical Education to provide CME for physicians.

**Credit Designation**

Hospital for Special Surgery designates this journal-based CME activity for a maximum of *1.0 AMA PRA Category 1 Credit(s)™*. Physicians should claim only the credit commensurate with the extent of their participation in the activity.

**Commercial Support**

This journal-based activity did not receive commercial support.

**Financial Disclosure**

In accordance with the Accreditation Council for Continuing Medical Education’s Standards for Commercial Support, all CME providers are required to disclose to the activity audience the relevant financial relationships of the planners, teachers, and authors involved in the development of CME content. An individual has a relevant financial relationship if he or she has a financial relationship in any amount occurring in the last 12 months with a commercial interest whose products or services are discussed in the CME activity content over which the individual has control.

It is the policy of Hospital for Special Surgery to request all financial relationships that Activity Directors, planning committee members, presenters, authors, and staff have with commercial interests but to disclose to the activity audience only the relevant financial relationships.

**Activity Directors’ Disclosures**

**Charles N. Cornell, MD,** has disclosed no relevant financial relationships.

**Joy Jacobson, MFA,** has disclosed no relevant financial relationships.

**Planning Committee Disclosures**

**Charles N. Cornell, MD,** has disclosed no relevant financial relationships.

**Joseph D. Giordano** has disclosed no relevant financial relationships.

**Adam Schumaier, MD,** has disclosed no relevant financial relationships.

**Amy R. Stair, MS,** has disclosed no relevant financial relationships.

**Faculty Disclosure**

**Adam Schumaier, MD,** has disclosed no relevant financial relationships.

**OCME/CME Committee Disclosure**

Hospital for Special Surgery Office of CME Staff and CME Committee members have no relevant financial relationships to disclose regarding this activity.

**Instructions for Post-test, Course Evaluation, and CME Credit**

In order to earn CME credit, you must complete an online post-test and evaluation following the completion of this activity. There is a passing requirement of 100%. Once you complete the post-test and subsequent evaluation, a certificate will be available for you to print.

For questions related to the post-test and subsequent evaluation, please contact *HSS Journal*’s managing editor, Joy Jacobson, at jacobsonj@hss.edu or 646-797-8509.

*To complete the activity for CME Credit:*

1. Go to the *HSS Journal* homepage at www.springer.com/hss.

2. Click on the “CME and Free-to-Access Articles” Tab.

3. Click on the article title to view the full-text PDF article.

4. After you have reviewed the article click on “Complete the Current CME Test Online” to complete the test.

5. Once you have passed the post-test with a score of 100%, you will be able to complete the evaluation. You will then be able to print your CME certificate.

## Introduction

Rotator cuff tears are a common cause of shoulder disability [30]. Studies have estimated that the prevalence of full-thickness rotator cuff tears in the general population ranges from 15 to 22% [26, 36] and steadily increases beyond the fifth decade of life [25]. The pathogenesis and natural history of a rotator cuff tear is largely unknown [9], and it is difficult to predict which patients will develop symptoms [33]. However, some patients with massive and irreparable tears can develop cuff tear arthropathy (CTA), which makes management more challenging. The clinical condition of CTA was first described in the nineteenth century [1, 38], and the term *cuff tear arthropathy* was first reported by Neer et al. in 1983 [28]. CTA is characterized by chronic rotator cuff insufficiency that can lead to migration of the humeral head, pain, impaired range of motion, and degenerative changes of articular structures [20, 33].

Several radiographic and clinical factors can aid in the evaluation of CTA, and multiple radiographic classifications have been described. The specific radiographic features evaluated vary with each classification [10, 15, 37, 39], but in general, the classifications assess humeral head migration and pathologic bony changes. In addition to radiographic assessment of the shoulder, clinical factors that can influence the management of CTA include the patient’s age, symptom severity, activity level, and range of motion [27]. Deciding how to manage patients can be difficult. Symptoms and activity level do not always correlate with the severity [8] and prevalence of tears [25, 36], and arthritic radiographic findings do not always correlate with symptoms [17, 32].

It is not clear which factors are most important to shoulder surgeons when deciding among non-operative management, arthroscopic treatment, or joint replacement for patients with CTA. The purposes of this study were to (1) determine what factors are most important to shoulder surgeons when making clinical decisions and (2) determine the inter- and intraobserver reliability of the Seebauer and Hamada radiographic grading systems. Our hypothesis was that symptoms and radiographic grade would have the strongest impact on clinical decision making and that the two grading systems would have similar inter- and intraobserver reliability.

## Methods

We designed a case-based survey that would be sent to five practicing, fellowship-trained shoulder surgeons in August and September of 2017. The survey consisted of 108 cases of simulated patients “presenting” with CTA that were accompanied by shoulder X-rays. Each simulated patient had a different combination of age (30, 45, or 65 years of age), symptoms (mild, moderate, or severe), activity level (low, moderate, or high), and range of motion (functional or non-functional). The ages of 30, 45, and 65 years were selected because they were spaced out sufficiently to detect the effects of age on treatment decisions.

The vignettes for activity level and symptoms included the following. A “low” activity level indicated a patient who was inactive, with very low demand on the shoulder; “moderate” indicated a patient who worked a desk job and enjoyed playing recreational basketball and lifting weights; and “high” indicated a patient who was very active, a manual laborer. Symptoms were “mild” if they arose only during strenuous activities, “moderate” if they limited most activities, and “severe” if they were constant.

To test each combination of age, activity level, symptoms, and range of motion, 54 cases were required. Each of the 54 cases was then paired with either a low-grade or a high-grade radiograph—the radiographic grade based on the degree of humeral head migration and pathologic bony changes—leading to 108 cases. Two authors (A.P.S. and B.M.G.) agreed upon the radiographic grades prior to assigning the radiographs to patient cases. The survey was sent in two parts, and the 54 radiographs from the first survey were also used in the second survey to assess the intraobserver reliability of the grading systems. The second survey was sent 1 month after the first survey to allow for appropriate washout. Each patient factor was equally represented in the survey, and every possible combination of factors was used exactly once. The case number of 108 is derived from three age levels, three activity levels, three symptom levels, two radiographic levels, and two range-of-motion levels (3 × 3 × 3 × 2 × 2 = 108). Dividing the survey into two parts allowed us to minimize responder burden and assess the intraobserver reliability of the grading systems. There was a true anteroposterior (AP) Grashey view and an axillary view radiograph of the glenohumeral joint provided for each case.

We included three questions with each case. The first two asked the surgeon to grade the radiographs per Seebauer [39] and then Hamada [15] (Table 1). The third question asked the surgeon to choose a treatment for the patient from four treatment choices: (1) non-operative treatment (medications, injections, physical therapy, or watchful waiting), (2) arthroscopic treatment, (3) hemiarthroplasty, and (4) reverse total shoulder arthroplasty (RTSA). The type of arthroscopic treatment was not specified because an evaluation of the various techniques was outside the scope of this study. Throughout the survey, the respondent was asked to assume that the rotator cuff was torn and irreparable and that each patient had achieved limited success with a corticosteroid injection and physical therapy. The cases were randomized, so that each surgeon reviewed the cases in a different order. The full survey was designed in SurveyMonkey (San Mateo, CA, USA) (see Online Resources 1 and 2).

**Table 1:** Seebauer and Hamada radiographic grading systems for cuff tear arthropathy

Seebauer	
Type 1A	Centered, stable: minimal superior migration, coracoacromial (C-A) arch acetabularization and femoralization of humeral head
Type 1B	Centered, medialized: minimal superior migration, medial glenoid erosion, C-A arch acetabularization and femoralization of humeral head
Type 2A	Decentered, limited stable: superior migration, superior-medial erosion, extensive C-A arch acetabularization and femoralization of humeral head
Type 2B	Decentered, unstable: anterior-superior escape, absent stabilization, C-A arch and anterior structures deficient
Hamada	
Grade 1	Acromio-humeral interval (AHI) > 6 mm
Grade 2	AHI 5 mm or less
Grade 3	Grade 2 with acetabularization of acromion
Grade 4	Grade 3 with narrowing of glenohumeral joint
Grade 5	Grade 4 with bony destruction and humeral head collapse

All five surgeons completing the 108 cases would result in a total of 540 responses. This number provides greater than 90% power to detect a moderate correlation (*r* = 0.4), with an alpha of 0.05 [6]. A Spearman’s correlation and *χ*^2^ test were performed to assess how the surgeon’s treatment decisions were related to each simulated patient’s age, activity level, symptoms, radiographic grade, and range of motion. The treatments were converted to an ordinal scale based on progression of care (i.e., non-operative = 1, arthroscopy = 2, hemiarthroplasty = 3, and RTSA = 4). The treatments were then individually correlated with age, activity level, symptom, radiographic grade, and range of motion. An additional sub-analysis was done on those cases for which surgeons chose an operative intervention to correlate patient factors with the type of operation chosen. A correlation of 0 suggests no association, and a correlation of 1 suggests perfect association [18]. In this study, we interpreted the Spearman’s correlations based on the effect size values suggested by Cohen [6]: weak, less than 0.3; moderate, 0.3 to 0.5; strong, more than 0.5.

The inter- and intraobserver reliability of the Seebauer and Hamada grading systems was calculated based on the grades assigned by the five survey respondents using an intraclass correlation (ICC). The strength of the observer reliability was interpreted according to Cicchetti [5]: poor, less than 0.4; fair, 0.40 to 0.59; good, 0.60 to 0.74; excellent, more than 0.75. All statistical tests were performed using R, version 3.4.0 [31] (R Foundation for Statistical Computing, Vienna, Austria), with RStudio, version 1.0.153 [34] (RStudio Inc., Boston, MA, USA).

## Results

All five surgeons completed the first and second surveys; hence, 540 cases were completed. The strength of the correlations for the factors ranged from none to moderate. The strongest factor was symptoms (0.45), followed by Hamada grade (0.38), patient age (0.34), and Seebauer type (0.29). Activity level and range of motion did not reach significance (Table 2). This indicates that more severe symptoms, older age, and higher Seebauer and Hamada grades were more likely to result in progression of care, while range of motion and activity level did not have a statistically significant effect. Similarly, the *χ*^2^ test for symptoms, age, and Seebauer or Hamada grade reached significance, while range of motion and activity level did not.

**Table 2:** Correlation between treatment and patient-specific factors

	Spearman’s *r* (95% CI)	*p* value	Interpretation
All cases			
Symptoms	0.45 (0.37 to 0.51)	*p <* 0.001	Moderate to strong
Hamada grade	0.38 (0.30 to 0.45)	*p <* 0.001	Moderate
Age	0.34 (0.26 to 0.42)	*p <* 0.001	Weak to moderate
Seebauer type	0.29 (0.21 to 0.37)	*p <* 0.001	Weak to moderate
Activity level	− 0.05 (− 0.13 to 0.04)	*p =* 0.25	None
Range of motion	− 0.04 (− 0.13 to 0.04)	*p =* 0.30	None
Sub-analysis of operative cases			
Hamada grade	0.56 (0.49 to 0.62)	*p <* 0.001	Moderate to strong
Age	0.51 (0.43 to 0.59)	*p <* 0.001	Moderate to strong
Seebauer type	0.46 (0.37 to 0.53)	*p <* 0.001	Moderate to strong
Activity level	− 0.13 (− 0.23 to − 0.04)	*p =* 0.007	Weak
Symptoms	− 0.01(− 0.12 to 0.09)	*p =* 0.86	None
Range of motion	0.03 (− 0.07 to 0.12)	*p =* 0.58	None

**Fig. 1:**
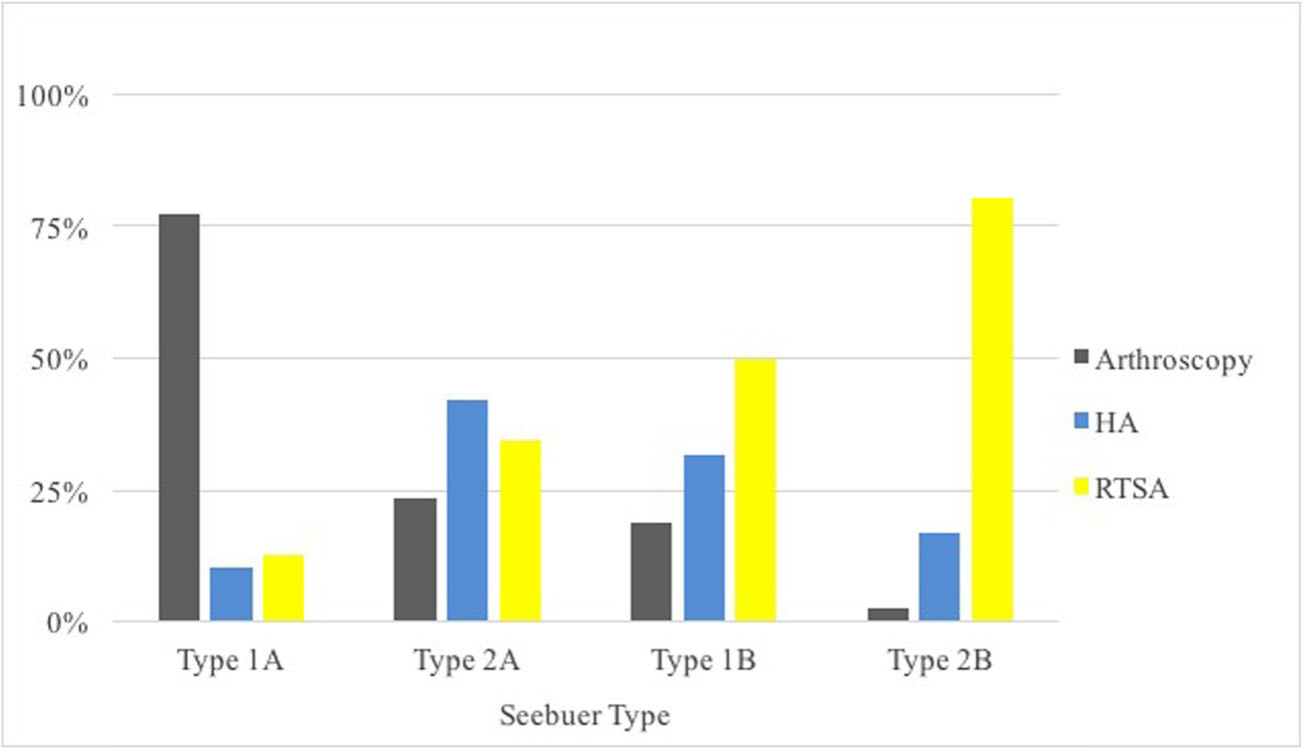
Treatments chosen for cases in which a surgical option was selected (*n* = 404), grouped based on patients’ radiographic disease severity (Seebauer type). *HA* hemiarthroplasty, *RTSA* reverse total shoulder arthroplasty

**Fig. 2:**
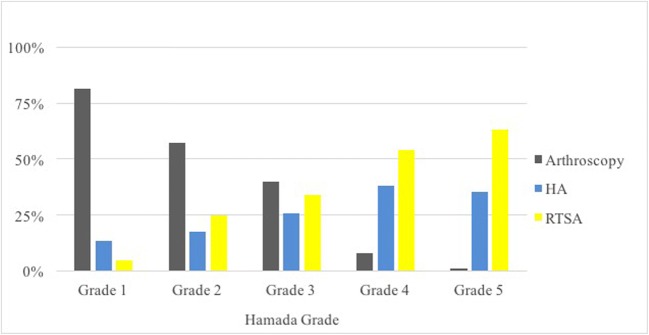
Treatments chosen for cases in which a surgical option was selected (*n* = 404), grouped based on patients’ radiographic disease severity (Hamada grade). *HA *hemiarthroplasty, *RTSA* reverse total shoulder arthroplasty

**Fig. 3:**
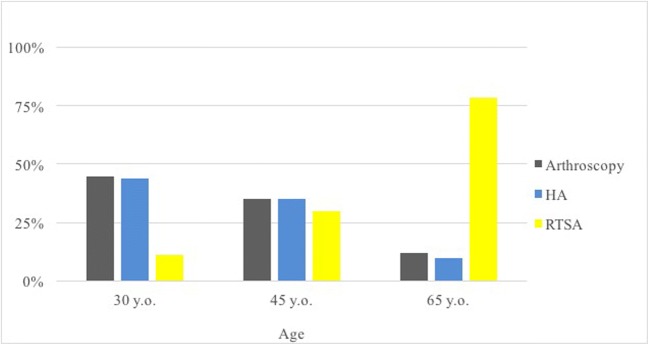
Treatments chosen for cases in which a surgical option was selected (*n* = 404), grouped based on patients’ age. *HA* hemiarthroplasty, *RTSA* reverse total shoulder arthroplasty

In the sub-analysis of cases in which the surgeons chose to pursue operative treatment, the strength of the correlations ranged from none to strong. The strongest factor was Hamada grade (0.56), followed by patient age (0.51), Seebauer type (0.46), and activity level (− 0.13). Patient symptoms and range of motion did not reach significance (Table 2, Figs. 1, 2, and 3). This indicates that radiographic grade and age were associated with progression of surgical treatments while symptoms, activity level, and range of motion were not.

In total, 540 radiographs were graded. The distribution of radiographic grades was as follows: Seebauer type 1A (114), Seebauer type 1B (158), Seebauer type 2A (213), Seebauer type 2B (55); Hamada grade 1 (89), Hamada grade 2 (92), Hamada grade 3 (71), Hamada grade 4 (198), Hamada grade 5 (90). The interobserver reliabilities of the grading systems were fair to good: Seebauer type (0.59), Hamada grade (0.58). Similarly, the intraobserver reliabilities were fair to good: Seebauer type (0.63), Hamada grade (0.65). The interobserver agreement for treatment decisions was poor to fair (0.44) (Table 3).

**Table 3:** Intra- and interobserver reliability

	ICC (95% CI)	*p* value	Interpretation
Interobserver reliability			
Seebauer type	0.59 (0.50 to 0.67)	*p <* 0.001	Fair to good
Hamada grade	0.58 (0.48 to 0.67)	*p <* 0.001	Fair to good
Treatment	0.44 (0.38 to 0.56)	*p <* 0.001	Poor to good
Intraobserver reliability			
Seebauer type	0.63 (0.56 to 0.70)	*p <* 0.001	Fair to good
Hamada grade	0.65 (0.58 to 0.72)	*p <* 0.001	Fair to good

*ICC* intraclass correlation

## Discussion

CTA is a challenging condition to manage, and patients can present with significant pain and loss of function [28, 33]. Evaluation typically includes the patient’s activity level, range of motion, symptoms, age, and imaging findings [27]. Non-operative management, arthroscopy, and joint replacement can be effective treatments in properly selected patients [2, 9, 27]. The primary purpose of this study was to quantify how different patient factors affect the treatment decisions made by shoulder specialists. Our results suggest that patient symptoms, patient age, and radiographic findings have the strongest association with clinical decision making; activity level and range of motion have a weaker association. Symptoms are the primary reason to treat any patient, and it was not surprising that this factor was most strongly correlated with treatment choice (*r* = .45). Interestingly, symptoms were not correlated with treatment choice in the sub-analysis of operative cases (*r* = − 0.01). This suggests that symptoms are an important indication for surgery, but the choice of operation is guided by other variables such as patient age and radiographic disease severity.

This study has several limitations. All respondents were practicing, fellowship-trained shoulder surgeons, and these results may not apply to other practices. Further, these opinions were based on information available in 2017 to 2018 and may change as treatment for CTA advances. The values of the various patient factors were arbitrarily chosen and do not reflect the true spectrum of patient presentations. This is especially true regarding range of motion, which we considered only as either functional or non-functional; however, the overall goal was to distinguish the effect of pseudoparalysis on clinical decision making. Further, several additional factors that could influence decision making, such as patient compliance, expectations, and access to health care resources, were not evaluated in this study. This could partially explain the variable agreement regarding treatment decisions. Additionally, there was a slight correlation between patient age and radiographic grade, which indicates either that higher-grade radiographs were inadvertently assigned to the older patients or that surgeons’ grading was influenced by the patients’ age. The correlation was minimal, but this suggests that the true effect of age and radiographic grade is slightly smaller than the reported values. Despite these limitations, the number of cases evaluated was large (540), sufficient power was achieved, two different statistical measures of association were calculated, and the results were analyzed descriptively. Additionally, the factors we evaluated were equally weighted and are frequently mentioned in the literature as important in evaluating patients with CTA. Finally, although intraobserver agreement of the radiographic grading was calculated, the intraobserver reliability of the treatment decisions was not measured.

Age and radiographic grade were significantly associated with the choice of treatment in all cases, which suggests these factors are important for both deciding to operate and choosing an operation. Arthroscopy and hemiarthroplasty were equally favored in the younger age groups, and arthroscopy was the clear surgery of choice in lower-grade arthritis. Arthroscopy is a useful joint-preserving technique that has demonstrated good results in earlier radiographic stages of CTA [4, 23]. Retrospective reviews of 31 and 41 patients with irreparable cuff tears by Liem et al. [23] and Klinger et al. [22] found significantly improved functional scores and decreased pain following arthroscopic debridement; 24 and 17 patients, respectively, underwent additional biceps tenotomy at the time of debridement. Similarly, a review of 68 patients by Boileau et al. found that biceps tenotomy or tenodesis significantly improved pain and functional scores in patients with irreparable rotator cuff tears and associated biceps tendon pathology [4]. More recently, superior capsular reconstruction (SCR) has garnered attention as an arthroscopic joint-preserving technique. It has demonstrated considerable improvements in pain and range of motion in short-to-mid-duration studies of patients with irreparable rotator cuff tears [7, 24]. A more thorough review of SCR was recently written by Hartzler and Burkhart [16].

As patient age and radiographic grade increased, RTSA was progressively favored over hemiarthroplasty and arthroscopy. Hemiarthroplasty was likely favored in the younger patients because it is thought to preserve the glenoid. A review by Feeley et al. [11] points out several studies that have demonstrated satisfactory long-term results in a majority of patients with CTA treated with hemiarthroplasty [12, 14, 35, 39, 41, 42]. However, concerns persist for continued bone loss and humeral head migration [11, 12, 35], and so hemiarthroplasty is typically limited to patients with an intact coracoacromial arch [27, 42]. Loss of coracoacromial arch integrity is a feature of higher-grade CTA, which likely explains why recommendations for RTSA were strongly associated with Seebauer and Hamada grades. The RTSA has shown promising results [3, 13, 37, 40], but concerns for complications [11] may include scapular notching [3, 37], implant failure [13], and re-operation [40]. For these reasons, most authors recommend RTSA only in elderly and low-demand patients [3, 11, 37], which is similar to our findings (RTSA recommended in 8% of 30-year-olds and 62% of 65-year-olds).

Activity level is thought to be an important factor for evaluating CTA [9, 11]. Surprisingly, this was one of the least important factors in our study with an *r* of – 0.05. There was a slight negative correlation with treatment recommendations in the sub-analysis of operative cases (*r* = − 0.13), indicating that more active patients were slightly more likely to be treated with arthroscopy or hemiarthroplasty than with RTSA. This could be due to concerns for implant complications [9, 11] and component stability [29] in highly active individuals.

Range of motion is thought to be an important factor to evaluate because arthroscopy and hemiarthroplasty are less likely than RTSA to restore range of motion [33]. Despite the statistical significance of the *χ*^2^ test (*p* = 0.03), there was no practically significant difference in the treatments chosen between patients who did and did not have a functional range of motion, especially in comparison to the other factors evaluated. It is not clear why range of motion was a minor factor, but it could be due to limitations in the study design and the fact that tendon transfer was not offered as an option.

There are several classification systems that have been used for CTA, including the Seebauer [39], Hamada [15], Favard [10], and Sirveaux [37] classifications. To limit the effects of responder burden, we designed our study to evaluate only two classifications, the Seebauer and Hamada systems, because they have been used in similar studies [19, 21] and evaluate similar features. The Sirveaux classification primarily is based on glenoid changes, and so it was felt that comparison with the other systems would not be fair [37]. The Favard classification was originally designed to distinguish between CTA (group 1), glenohumeral joint narrowing (group 2), and rheumatologic shoulders (group 3) [10], so it was thought to be inapplicable to this study.

Our results demonstrated that the Seebauer and Hamada systems had very similar inter- and intraobserver reliability (fair to good). Other studies that have evaluated the reliability of these systems reported moderate agreement [19, 21]; however, direct comparison of the numbers is difficult because it is not clear whether these studies used unweighted or weighted κ statistics. Despite the variability in interpreting the radiographic grades, both systems had significant associations with the choice of treatment. The Hamada grading system had a stronger correlation than the Seebauer system, but there is enough overlap of the confidence intervals that it would be difficult to recommend one over the other. Additionally, the correlation measure is likely biased in favor of the Hamada grading system; Hamada grades have a more ordinal nature than Seebauer types. In fact, when reviewing the *χ*^2^ frequency tables closely (Table 4), the Seebauer system better discriminated between the choice of hemiarthroplasty versus RTSA. Based on the overall results, the Seebauer and Hamada systems have similar clinical utility. Future research should focus on the development of a more robust and concise grading system that allows for reliable treatment prediction and stronger observer agreement.

**Table 4:** Frequency of treatments grouped by patient factor (*χ*^2^ test)

Hamada grade (*p <* 0.001)					
	Grade 1 (%)	Grade 2 (%)	Grade 3 (%)	Grade 4 (%)	Grade 5 (%)
Non-operative	33	26	30	23	19
Arthroscopy	55	42	28	6	1
HA	9	13	18	29	29
RTSA	3	18	24	42	51
Seebauer type (*p <* 0.001)					
	Type 1A (%)	Type 1B (%)	Type 2A (%)	Type 2B (%)	
Non-operative	31	25	23	25	
Arthroscopy	54	18	15	2	
HA	7	32	24	13	
RTSA	9	26	38	60	
Age (*p <* 0.001)					
	30 years (%)	45 years (%)	65 years (%)		
Non-operative	29	26	21		
Arthroscopy	32	26	9		
HA	31	26	8		
RTSA	8	22	62		
Activity (*p =* 0.148)					
	Low (%)	Moderate (%)	High (%)		
Non-operative	26	28	22		
Arthroscopy	18	22	27		
HA	19	21	26		
RTSA	37	29	26		
Symptoms (*p <* 0.001)					
	Mild (%)	Moderate (%)	Severe (%)		
Non-operative	65	7	4		
Arthroscopy	13	21	33		
HA	8	34	23		
RTSA	13	39	40		
Range of motion (*p =* 0.03)					
	Functional (%)	Non-functional (%)			
Non-operative	29	22			
Arthroscopy	19	26			
HA	24	19			
TSA	29	33			

*HA* hemiarthroplasty, *RTSA* reverse total shoulder arthroplasty

In conclusion, in the evaluation of CTA, patient symptoms, radiographic grade, and patient age were the factors most strongly associated with the clinical decision making of shoulder specialists. In the sub-analysis of cases treated surgically, the factors most strongly associated with the choice of operation were radiographic grade (disease severity) and patient age. Activity level and range of motion had only a minimal effect on the management decisions. Additionally, we found that the Seebauer and Hamada classifications had fair to good inter- and intraobserver reliability. This study quantified several important factors in the evaluation of patients with CTA, but there was only fair agreement among shoulder specialists. This could be due to study limitations or differences in treatment philosophies; it highlights the lack of evidence-based guidelines for managing this often-challenging condition.

## Electronic supplementary material

ESM 1 (PDF 1917 kb)

ESM 2 (PDF 1409 kb)

## References

[1] Adams R (1873). A Treatise on Rheumatic Gout or Chronic Rheumatic Arthritis of All the Joints.

[2] Aumiller WD, Kleuser TM (2015). Diagnosis and treatment of cuff tear arthropathy. JAAPA..

[3] Boileau P, Watkinson DJ, Hatzidakis AM, Balg F (2005). Grammont reverse prosthesis: design, rationale, and biomechanics. J Shoulder Elbow Surg..

[4] Boileau P, Baqué F, Valerio L, Ahrens P, Chuinard C, Trojani C (2007). Isolated arthroscopic biceps tenotomy or tenodesis improves symptoms in patients with massive irreparable rotator cuff tears. J Bone Joint Surg Am..

[5] Cicchetti DV (1994). Guidelines, criteria, and rules of thumb for evaluating normed and standardized assessment instruments in psychology. Psychol Assess..

[6] Cohen J (1992). A power primer. Psychol Bull..

[7] Denard PJ, Brady PC, Adams CR, Tokish JM, Burkhart SS (2018). Preliminary results of arthroscopic superior capsule reconstruction with dermal allograft. Arthroscopy..

[8] Dunn WR, Kuhn JE, Sanders R (2014). Symptoms of pain do not correlate with rotator cuff tear severity: a cross-sectional study of 393 patients with a symptomatic atraumatic full-thickness rotator cuff tear. J Bone Joint Surg Am..

[9] Ecklund KJ, Lee TQ, Tibone J, Gupta R (2007). Rotator cuff tear arthropathy. J Am Acad Orthop Surg..

[10] Favard L, Lautmann S, Clement P (1999). Osteoarthritis with massive rotator cuff-tear: the limitation of its current definitions. Shoulder Arthroplasty.

[11] Feeley BT, Gallo RA, Craig EV (2009). Cuff tear arthropathy: current trends in diagnosis and surgical management. J Shoulder Elbow Surg..

[12] Field LD, Dines DM, Zabinski SJ, Warren RF (1997). Hemiarthroplasty of the shoulder for rotator cuff arthropathy. J Shoulder Elbow Surg..

[13] Frankle M, Levy JC, Pupello D (2006). The reverse shoulder prosthesis for glenohumeral arthritis associated with severe rotator cuff deficiency. a minimum two-year follow-up study of sixty patients surgical technique. J Bone Joint Surg Am..

[14] Goldberg NS, Collins FS (1991). The hunt for the neurofibromatosis gene. Arch Dermatol..

[15] Hamada K, Fukuda H, Mikasa M, Kobayashi Y. Roentgenographic findings in massive rotator cuff tears. A long-term observation. *Clin Orthop*. 1990;(254):92–96. 2323152

[16] Hartzler RU, Burkhart SS (2017). Superior capsular reconstruction. Orthopedics..

[17] Holzer N, Salvo D, Marijnissen ACA (2015). Radiographic evaluation of posttraumatic osteoarthritis of the ankle: the Kellgren-Lawrence scale is reliable and correlates with clinical symptoms. Osteoarthr Cartil..

[18] Hung M, Bounsanga J, Voss MW (2017). Interpretation of correlations in clinical research. Postgrad Med..

[19] Iannotti JP, McCarron J, Raymond CJ (2010). Agreement study of radiographic classification of rotator cuff tear arthropathy. J Shoulder Elbow Surg..

[20] Jensen KL, Williams GR, Russell IJ, Rockwood CA (1999). Rotator cuff tear arthropathy. J Bone Joint Surg Am..

[21] Kappe T, Cakir B, Reichel H, Elsharkawi M (2011). Reliability of radiologic classification for cuff tear arthropathy. J Shoulder Elbow Surg..

[22] Klinger HM, Spahn G, Baums MH, Steckel H (2005). Arthroscopic debridement of irreparable massive rotator cuff tears: a comparison of debridement alone and combined procedure with biceps tenotomy. Acta Chir Belg..

[23] Liem D, Lengers N, Dedy N, Poetzl W, Steinbeck J, Marquardt B (2008). Arthroscopic debridement of massive irreparable rotator cuff tears. Arthroscopy..

[24] Mihata T, Lee TQ, Watanabe C (2013). Clinical results of arthroscopic superior capsule reconstruction for irreparable rotator cuff tears. Arthroscopy..

[25] Milgrom C, Schaffler M, Gilbert S, van Holsbeeck M (1995). Rotator-cuff changes in asymptomatic adults. The effect of age, hand dominance and gender. J Bone Joint Surg Br..

[26] Minagawa H, Yamamoto N, Abe H (2013). Prevalence of symptomatic and asymptomatic rotator cuff tears in the general population: from mass-screening in one village. J Orthop..

[27] Nam D, Maak TG, Raphael BS, Kepler CK, Cross MB, Warren RF (2012). Rotator cuff tear arthropathy: evaluation, diagnosis, and treatment: AAOS exhibit selection. J Bone Joint Surg Am..

[28] Neer CS, Craig EV, Fukuda H (1983). Cuff-tear arthropathy. J Bone Joint Surg Am..

[29] Nicholson GP, Strauss EJ, Sherman SL (2011). Scapular notching: Recognition and strategies to minimize clinical impact. Clin Orthop..

[30] Oh LS, Wolf BR, Hall MP, Levy BA, Marx RG (2007). Indications for rotator cuff repair: A systematic review. Clin Orthop..

[31] R Core Team (2017). R: A Language and Environment for Statistical Computing.

[32] Riddle DL, Jiranek WA (2015). Knee osteoarthritis radiographic progression and associations with pain and function prior to knee arthroplasty: a multicenter comparative cohort study. Osteoarthr Cartil..

[33] Rockwood CA (2009). The Shoulder.

[34] RStudio Team (2016). RStudio: Integrated Development Environment for R.

[35] Sanchez-Sotelo J, Cofield RH, Rowland CM (2001). Shoulder hemiarthroplasty for glenohumeral arthritis associated with severe rotator cuff deficiency. J Bone Joint Surg Am..

[36] Sher JS, Uribe JW, Posada A, Murphy BJ, Zlatkin MB (1995). Abnormal findings on magnetic resonance images of asymptomatic shoulders. J Bone Joint Surg Am..

[37] Sirveaux F, Favard L, Oudet D, Huquet D, Walch G, Molé D (2004). Grammont inverted total shoulder arthroplasty in the treatment of glenohumeral osteoarthritis with massive rupture of the cuff. Results of a multicentre study of 80 shoulders. J Bone Joint Surg Br..

[38] Smith RW (1853). Observations upon chronic rheumatic arthritis of the shoulder. Dublin Q J Med Sci..

[39] Visotsky JL, Basamania C, Seebauer L, Rockwood CA, Jensen KL (2004). Cuff tear arthropathy: pathogenesis, classification, and algorithm for treatment. J Bone Joint Surg Am..

[40] Werner CML, Steinmann PA, Gilbart M, Gerber C (2005). Treatment of painful pseudoparesis due to irreparable rotator cuff dysfunction with the Delta III reverse-ball-and-socket total shoulder prosthesis. J Bone Joint Surg Am..

[41] Williams GR, Rockwood CA (1996). Hemiarthroplasty in rotator cuff-deficient shoulders. J Shoulder Elbow Surg..

[42] Zuckerman JD, Scott AJ, Gallagher MA (2000). Hemiarthroplasty for cuff tear arthropathy. J Shoulder Elbow Surg..

