# Supplementary material for: Quantifying the Impact of Patient-Specific Factors and Disease Severity on Clinical Decision Making in Cuff Tear Arthropathy: A Case-Based Survey
Source: HSS J. 2019 Jul 5;15(3):276–85. doi: 10.1007/s11420-019-09695-x (PMC6778159; doi:10.1007/s11420-019-09695-x)
Supplement: Supplementary file 1 — (PDF 1917 kb) [file 11420_2019_9695_MOESM1_ESM.pdf]

Welcome to Our Survey

This survey contains 54 randomized, short clinical vignettes describing a patient who is presenting with signs and symptoms of cuff tear arthropathy. Assumptions are listed at the top of each page.

Each vignette has the same 3 questions:
1) Grade the radiographs according to Seebauer
2) Grade the radiographs according to Hamada
3) Select a treatment

Primary Objectives:
-Determine the demographics, signs, symptoms, and radiographic features that impact clinical decision making
-Determine 1) the reliability of the Seebauer and Hamada grading systems 2) whether or not the grading systems correlate with clinical decision making

Thank you for your time!

Case 1

An active 30 y.o. who works as a manual laborer presents with mild signs and symptoms (only during strenuous activities)

* 1. Please grade the radiographs according to Seebauer

- ◯ **Type 1A-Centered, Stable:** minimal superior migration, C-A arch acetabularization and femoralization of humeral head
- ◯ **Type 1B-Centered, Medialized:** minimal superior migration, medial glenoid erosion, C-A arch acetabularization and femoralization of humeral head
- ◯ **Type 2A-Decentered, Limited Stable:** superior migration, superior-medial erosion, extensive C-A arch acetabularization and femoralization of humeral head
- ◯ **Type 2B-Decentered, Unstable:** anterior-superior escape, absent stabilization, C-A arch and anterior structures deficient

* 2. Please grade the radiographs according to Hamada

- ◯ **Grade 1:** acromio-humeral interval (AHI) > 6mm
- ◯ **Grade 2:** acromio-humeral interval (AHI) 5mm or less
- ◯ **Grade 3:** grade 2 with acetabularization of acromion
- ◯ **Grade 4:** grade 3 with narrowing of gleno-humeral joint
- ◯ **Grade 5:** grade 4 with bony destruction and humeral head collapse

* 3. Please select a treatment

- ◯ non-operative (includes medications, injections, physical therapy, or watchful waiting)
- ◯ arthroscopic treatment
- ◯ hemiarthroplasty
- ◯ reverse shoulder arthroplasty

Case 2

A 30 y.o. who works a desk job and enjoys playing rec basketball and lifting weights presents with mild signs and symptoms (only during strenuous activities)

* 4. Please grade the radiographs according to Seebauer

- **Type 1A-Centered, Stable:** minimal superior migration, C-A arch acetabularization and femoralization of humeral head
- **Type 1B-Centered, Medialized:** minimal superior migration, medial glenoid erosion, C-A arch acetabularization and femoralization of humeral head
- **Type 2A-Decentered, Limited Stable:** superior migration, superior-medial erosion, extensive C-A arch acetabularization and femoralization of humeral head
- **Type 2B-Decentered, Unstable:** anterior-superior escape, absent stabilization, C-A arch and anterior structures deficient

* 5. Please grade the radiographs according to Hamada

- **Grade 1:** acromio-humeral interval (AHI) > 6mm
- **Grade 2:** acromio-humeral interval (AHI) 5mm or less
- **Grade 3:** grade 2 with acetabularization of acromion
- **Grade 4:** grade 3 with narrowing of gleno-humeral joint
- **Grade 5:** grade 4 with bony destruction and humeral head collapse

* 6. Please select a treatment

- non-operative (includes medications, injections, physical therapy, or watchful waiting)
- arthroscopic treatment
- hemiarthroplasty
- reverse shoulder arthroplasty

Case 3

An inactive 30 y.o. who is very low demand with their shoulder presents with mild signs and symptoms (only during strenuous activities)

* 7. Please grade the radiographs according to Seebauer

- **Type 1A-Centered, Stable:** minimal superior migration, C-A arch acetabularization and femoralization of humeral head
- **Type 1B-Centered, Medialized:** minimal superior migration, medial glenoid erosion, C-A arch acetabularization and femoralization of humeral head
- **Type 2A-Decentered, Limited Stable:** superior migration, superior-medial erosion, extensive C-A arch acetabularization and femoralization of humeral head
- **Type 2B-Decentered, Unstable:** anterior-superior escape, absent stabilization, C-A arch and anterior structures deficient

* 8. Please grade the radiographs according to Hamada

- **Grade 1:** acromio-humeral interval (AHI) > 6mm
- **Grade 2:** acromio-humeral interval (AHI) 5mm or less
- **Grade 3:** grade 2 with acetabularization of acromion
- **Grade 4:** grade 3 with narrowing of gleno-humeral joint
- **Grade 5:** grade 4 with bony destruction and humeral head collapse

* 9. Please select a treatment

- non-operative (includes medications, injections, physical therapy, or watchful waiting)
- arthroscopic treatment
- hemiarthroplasty
- reverse shoulder arthroplasty

Case 4

An active 30 y.o. who works as a manual laborer presents with moderate signs and symptoms (predominant symptoms that limit most things)

*** 10. Please grade the radiographs according to Seebauer**

- ◯ **Type 1A-Centered, Stable:** minimal superior migration, C-A arch acetabularization and femoralization of humeral head
- ◯ **Type 1B-Centered, Medialized:** minimal superior migration, medial glenoid erosion, C-A arch acetabularization and femoralization of humeral head
- ◯ **Type 2A-Decentered, Limited Stable:** superior migration, superior-medial erosion, extensive C-A arch acetabularization and femoralization of humeral head
- ◯ **Type 2B-Decentered, Unstable:** anterior-superior escape, absent stabilization, C-A arch and anterior structures deficient

*** 11. Please grade the radiographs according to Hamada**

- ◯ **Grade 1:** acromio-humeral interval (AHI) > 6mm
- ◯ **Grade 2:** acromio-humeral interval (AHI) 5mm or less
- ◯ **Grade 3:** grade 2 with acetabularization of acromion
- ◯ **Grade 4:** grade 3 with narrowing of gleno-humeral joint
- ◯ **Grade 5:** grade 4 with bony destruction and humeral head collapse

*** 12. Please select a treatment**

- ◯ non-operative (includes medications, injections, physical therapy, or watchful waiting)
- ◯ arthroscopic treatment
- ◯ hemiarthroplasty
- ◯ reverse shoulder arthroplasty

Case 5

A 30 y.o. who works a desk job and enjoys playing rec basketball and lifting weights presents with moderate signs and symptoms (predominant symptoms that limit most things)

* 13. Please grade the radiographs according to Seebauer

- ○ **Type 1A-Centered, Stable:** minimal superior migration, C-A arch acetabularization and femoralization of humeral head
- ○ **Type 1B-Centered, Medialized:** minimal superior migration, medial glenoid erosion, C-A arch acetabularization and femoralization of humeral head
- ○ **Type 2A-Decentered, Limited Stable:** superior migration, superior-medial erosion, extensive C-A arch acetabularization and femoralization of humeral head
- ○ **Type 2B-Decentered, Unstable:** anterior-superior escape, absent stabilization, C-A arch and anterior structures deficient

* 14. Please grade the radiographs according to Hamada

- ○ **Grade 1:** acromio-humeral interval (AHI) > 6mm
- ○ **Grade 2:** acromio-humeral interval (AHI) 5mm or less
- ○ **Grade 3:** grade 2 with acetabularization of acromion
- ○ **Grade 4:** grade 3 with narrowing of gleno-humeral joint
- ○ **Grade 5:** grade 4 with bony destruction and humeral head collapse

* 15. Please select a treatment

- ○ non-operative (includes medications, injections, physical therapy, or watchful waiting)
- ○ arthroscopic treatment
- ○ hemiarthroplasty
- ○ reverse shoulder arthroplasty

Case 6

An inactive 30 y.o. who is very low demand with their shoulder presents with moderate signs and symptoms (predominant symptoms that limit most things)

* 16. Please grade the radiographs according to Seebauer

- ◯ **Type 1A-Centered, Stable:** minimal superior migration, C-A arch acetabularization and femoralization of humeral head
- ◯ **Type 1B-Centered, Medialized:** minimal superior migration, medial glenoid erosion, C-A arch acetabularization and femoralization of humeral head
- ◯ **Type 2A-Decentered, Limited Stable:** superior migration, superior-medial erosion, extensive C-A arch acetabularization and femoralization of humeral head
- ◯ **Type 2B-Decentered, Unstable:** anterior-superior escape, absent stabilization, C-A arch and anterior structures deficient

* 17. Please grade the radiographs according to Hamada

- ◯ **Grade 1:** acromio-humeral interval (AHI) > 6mm
- ◯ **Grade 2:** acromio-humeral interval (AHI) 5mm or less
- ◯ **Grade 3:** grade 2 with acetabularization of acromion
- ◯ **Grade 4:** grade 3 with narrowing of gleno-humeral joint
- ◯ **Grade 5:** grade 4 with bony destruction and humeral head collapse

* 18. Please select a treatment

- ◯ non-operative (includes medications, injections, physical therapy, or watchful waiting)
- ◯ arthroscopic treatment
- ◯ hemiarthroplasty
- ◯ reverse shoulder arthroplasty

Case 7

An active 30 y.o. who works as a manual laborer presents with severe signs and symptoms (constant)

* 19. Please grade the radiographs according to Seebauer

- ◯ **Type 1A-Centered, Stable:** minimal superior migration, C-A arch acetabularization and femoralization of humeral head
- ◯ **Type 1B-Centered, Medialized:** minimal superior migration, medial glenoid erosion, C-A arch acetabularization and femoralization of humeral head
- ◯ **Type 2A-Decentered, Limited Stable:** superior migration, superior-medial erosion, extensive C-A arch acetabularization and femoralization of humeral head
- ◯ **Type 2B-Decentered, Unstable:** anterior-superior escape, absent stabilization, C-A arch and anterior structures deficient

* 20. Please grade the radiographs according to Hamada

- ◯ **Grade 1:** acromio-humeral interval (AHI) > 6mm
- ◯ **Grade 2:** acromio-humeral interval (AHI) 5mm or less
- ◯ **Grade 3:** grade 2 with acetabularization of acromion
- ◯ **Grade 4:** grade 3 with narrowing of gleno-humeral joint
- ◯ **Grade 5:** grade 4 with bony destruction and humeral head collapse

* 21. Please select a treatment

- ◯ non-operative (includes medications, injections, physical therapy, or watchful waiting)
- ◯ arthroscopic treatment
- ◯ hemiarthroplasty
- ◯ reverse shoulder arthroplasty

Case 8

A 30 y.o. who works a desk job and enjoys playing rec basketball and lifting weights presents with severe signs and symptoms (constant)

* 22. Please grade the radiographs according to Seebauer

- ◯ **Type 1A-Centered, Stable:** minimal superior migration, C-A arch acetabularization and femoralization of humeral head
- ◯ **Type 1B-Centered, Medialized:** minimal superior migration, medial glenoid erosion, C-A arch acetabularization and femoralization of humeral head
- ◯ **Type 2A-Decentered, Limited Stable:** superior migration, superior-medial erosion, extensive C-A arch acetabularization and femoralization of humeral head
- ◯ **Type 2B-Decentered, Unstable:** anterior-superior escape, absent stabilization, C-A arch and anterior structures deficient

* 23. Please grade the radiographs according to Hamada

- ◯ **Grade 1:** acromio-humeral interval (AHI) > 6mm
- ◯ **Grade 2:** acromio-humeral interval (AHI) 5mm or less
- ◯ **Grade 3:** grade 2 with acetabularization of acromion
- ◯ **Grade 4:** grade 3 with narrowing of gleno-humeral joint
- ◯ **Grade 5:** grade 4 with bony destruction and humeral head collapse

* 24. Please select a treatment

- ◯ non-operative (includes medications, injections, physical therapy, or watchful waiting)
- ◯ arthroscopic treatment
- ◯ hemiarthroplasty
- ◯ reverse shoulder arthroplasty

Case 9

An inactive 30 y.o. who is very low demand with their shoulder presents with severe signs and symptoms (constant)

* 25. Please grade the radiographs according to Seebauer

- ◯ **Type 1A-Centered, Stable:** minimal superior migration, C-A arch acetabularization and femoralization of humeral head
- ◯ **Type 1B-Centered, Medialized:** minimal superior migration, medial glenoid erosion, C-A arch acetabularization and femoralization of humeral head
- ◯ **Type 2A-Decentered, Limited Stable:** superior migration, superior-medial erosion, extensive C-A arch acetabularization and femoralization of humeral head
- ◯ **Type 2B-Decentered, Unstable:** anterior-superior escape, absent stabilization, C-A arch and anterior structures deficient

* 26. Please grade the radiographs according to Hamada

- ◯ **Grade 1:** acromio-humeral interval (AHI) > 6mm
- ◯ **Grade 2:** acromio-humeral interval (AHI) 5mm or less
- ◯ **Grade 3:** grade 2 with acetabularization of acromion
- ◯ **Grade 4:** grade 3 with narrowing of gleno-humeral joint
- ◯ **Grade 5:** grade 4 with bony destruction and humeral head collapse

* 27. Please select a treatment

- ◯ non-operative (includes medications, injections, physical therapy, or watchful waiting)
- ◯ arthroscopic treatment
- ◯ hemiarthroplasty
- ◯ reverse shoulder arthroplasty

Case 10

An active 45 y.o. who works as a manual laborer presents with mild signs and symptoms (only during strenuous activities)

* 28. Please grade the radiographs according to Seebauer

- ◯ **Type 1A-Centered, Stable:** minimal superior migration, C-A arch acetabularization and femoralization of humeral head
- ◯ **Type 1B-Centered, Medialized:** minimal superior migration, medial glenoid erosion, C-A arch acetabularization and femoralization of humeral head
- ◯ **Type 2A-Decentered, Limited Stable:** superior migration, superior-medial erosion, extensive C-A arch acetabularization and femoralization of humeral head
- ◯ **Type 2B-Decentered, Unstable:** anterior-superior escape, absent stabilization, C-A arch and anterior structures deficient

* 29. Please grade the radiographs according to Hamada

- ◯ **Grade 1:** acromio-humeral interval (AHI) > 6mm
- ◯ **Grade 2:** acromio-humeral interval (AHI) 5mm or less
- ◯ **Grade 3:** grade 2 with acetabularization of acromion
- ◯ **Grade 4:** grade 3 with narrowing of gleno-humeral joint
- ◯ **Grade 5:** grade 4 with bony destruction and humeral head collapse

* 30. Please select a treatment

- ◯ non-operative (includes medications, injections, physical therapy, or watchful waiting)
- ◯ arthroscopic treatment
- ◯ hemiarthroplasty
- ◯ reverse shoulder arthroplasty

Case 11

A 45 y.o. who works a desk job and enjoys playing rec basketball and lifting weights presents with mild signs and symptoms (only during strenuous activities)

*** 31. Please grade the radiographs according to Seebauer**

- **Type 1A-Centered, Stable:** minimal superior migration, C-A arch acetabularization and femoralization of humeral head
- **Type 1B-Centered, Medialized:** minimal superior migration, medial glenoid erosion, C-A arch acetabularization and femoralization of humeral head
- **Type 2A-Decentered, Limited Stable:** superior migration, superior-medial erosion, extensive C-A arch acetabularization and femoralization of humeral head
- **Type 2B-Decentered, Unstable:** anterior-superior escape, absent stabilization, C-A arch and anterior structures deficient

*** 32. Please grade the radiographs according to Hamada**

- **Grade 1:** acromio-humeral interval (AHI) > 6mm
- **Grade 2:** acromio-humeral interval (AHI) 5mm or less
- **Grade 3:** grade 2 with acetabularization of acromion
- **Grade 4:** grade 3 with narrowing of gleno-humeral joint
- **Grade 5:** grade 4 with bony destruction and humeral head collapse

*** 33. Please select a treatment**

- non-operative (includes medications, injections, physical therapy, or watchful waiting)
- arthroscopic treatment
- hemiarthroplasty
- reverse shoulder arthroplasty

Case 12

An inactive 45 y.o. who is very low demand with their shoulder presents with mild signs and symptoms (only during strenuous activities)

* 34. Please grade the radiographs according to Seebauer

- **Type 1A-Centered, Stable:** minimal superior migration, C-A arch acetabularization and femoralization of humeral head
- **Type 1B-Centered, Medialized:** minimal superior migration, medial glenoid erosion, C-A arch acetabularization and femoralization of humeral head
- **Type 2A-Decentered, Limited Stable:** superior migration, superior-medial erosion, extensive C-A arch acetabularization and femoralization of humeral head
- **Type 2B-Decentered, Unstable:** anterior-superior escape, absent stabilization, C-A arch and anterior structures deficient

* 35. Please grade the radiographs according to Hamada

- **Grade 1:** acromio-humeral interval (AHI) > 6mm
- **Grade 2:** acromio-humeral interval (AHI) 5mm or less
- **Grade 3:** grade 2 with acetabularization of acromion
- **Grade 4:** grade 3 with narrowing of gleno-humeral joint
- **Grade 5:** grade 4 with bony destruction and humeral head collapse

* 36. Please select a treatment

- non-operative (includes medications, injections, physical therapy, or watchful waiting)
- arthroscopic treatment
- hemiarthroplasty
- reverse shoulder arthroplasty

Case 13

An active 45 y.o. who works as a manual laborer presents with moderate signs and symptoms (predominant symptoms that limit most things)

*** 37. Please grade the radiographs according to Seebauer**

- **Type 1A-Centered, Stable:** minimal superior migration, C-A arch acetabularization and femoralization of humeral head
- **Type 1B-Centered, Medialized:** minimal superior migration, medial glenoid erosion, C-A arch acetabularization and femoralization of humeral head
- **Type 2A-Decentered, Limited Stable:** superior migration, superior-medial erosion, extensive C-A arch acetabularization and femoralization of humeral head
- **Type 2B-Decentered, Unstable:** anterior-superior escape, absent stabilization, C-A arch and anterior structures deficient

*** 38. Please grade the radiographs according to Hamada**

- **Grade 1:** acromio-humeral interval (AHI) > 6mm
- **Grade 2:** acromio-humeral interval (AHI) 5mm or less
- **Grade 3:** grade 2 with acetabularization of acromion
- **Grade 4:** grade 3 with narrowing of gleno-humeral joint
- **Grade 5:** grade 4 with bony destruction and humeral head collapse

*** 39. Please select a treatment**

- non-operative (includes medications, injections, physical therapy, or watchful waiting)
- arthroscopic treatment
- hemiarthroplasty
- reverse shoulder arthroplasty

Case 14

A 45 y.o. who works a desk job and enjoys playing rec basketball and lifting weights presents with moderate signs and symptoms (predominant symptoms that limit most things)

*** 40. Please grade the radiographs according to Seebauer**

- ◯ **Type 1A-Centered, Stable:** minimal superior migration, C-A arch acetabularization and femoralization of humeral head
- ◯ **Type 1B-Centered, Medialized:** minimal superior migration, medial glenoid erosion, C-A arch acetabularization and femoralization of humeral head
- ◯ **Type 2A-Decentered, Limited Stable:** superior migration, superior-medial erosion, extensive C-A arch acetabularization and femoralization of humeral head
- ◯ **Type 2B-Decentered, Unstable:** anterior-superior escape, absent stabilization, C-A arch and anterior structures deficient

*** 41. Please grade the radiographs according to Hamada**

- ◯ **Grade 1:** acromio-humeral interval (AHI) > 6mm
- ◯ **Grade 2:** acromio-humeral interval (AHI) 5mm or less
- ◯ **Grade 3:** grade 2 with acetabularization of acromion
- ◯ **Grade 4:** grade 3 with narrowing of gleno-humeral joint
- ◯ **Grade 5:** grade 4 with bony destruction and humeral head collapse

*** 42. Please select a treatment**

- ◯ non-operative (includes medications, injections, physical therapy, or watchful waiting)
- ◯ arthroscopic treatment
- ◯ hemiarthroplasty
- ◯ reverse shoulder arthroplasty

Case 15

An inactive 45 y.o. who is very low demand with their shoulder presents with moderate signs and symptoms (predominant symptoms that limit most things)

* 43. Please grade the radiographs according to Seebauer

- ◯ **Type 1A-Centered, Stable:** minimal superior migration, C-A arch acetabularization and femoralization of humeral head
- ◯ **Type 1B-Centered, Medialized:** minimal superior migration, medial glenoid erosion, C-A arch acetabularization and femoralization of humeral head
- ◯ **Type 2A-Decentered, Limited Stable:** superior migration, superior-medial erosion, extensive C-A arch acetabularization and femoralization of humeral head
- ◯ **Type 2B-Decentered, Unstable:** anterior-superior escape, absent stabilization, C-A arch and anterior structures deficient

* 44. Please grade the radiographs according to Hamada

- ◯ **Grade 1:** acromio-humeral interval (AHI) > 6mm
- ◯ **Grade 2:** acromio-humeral interval (AHI) 5mm or less
- ◯ **Grade 3:** grade 2 with acetabularization of acromion
- ◯ **Grade 4:** grade 3 with narrowing of gleno-humeral joint
- ◯ **Grade 5:** grade 4 with bony destruction and humeral head collapse

* 45. Please select a treatment

- ◯ non-operative (includes medications, injections, physical therapy, or watchful waiting)
- ◯ arthroscopic treatment
- ◯ hemiarthroplasty
- ◯ reverse shoulder arthroplasty

Case 16

An active 45 y.o. who works as a manual laborer presents with severe signs and symptoms (constant)

* 46. Please grade the radiographs according to Seebauer

- ◯ **Type 1A-Centered, Stable:** minimal superior migration, C-A arch acetabularization and femoralization of humeral head
- ◯ **Type 1B-Centered, Medialized:** minimal superior migration, medial glenoid erosion, C-A arch acetabularization and femoralization of humeral head
- ◯ **Type 2A-Decentered, Limited Stable:** superior migration, superior-medial erosion, extensive C-A arch acetabularization and femoralization of humeral head
- ◯ **Type 2B-Decentered, Unstable:** anterior-superior escape, absent stabilization, C-A arch and anterior structures deficient

* 47. Please grade the radiographs according to Hamada

- ◯ **Grade 1:** acromio-humeral interval (AHI) > 6mm
- ◯ **Grade 2:** acromio-humeral interval (AHI) 5mm or less
- ◯ **Grade 3:** grade 2 with acetabularization of acromion
- ◯ **Grade 4:** grade 3 with narrowing of gleno-humeral joint
- ◯ **Grade 5:** grade 4 with bony destruction and humeral head collapse

* 48. Please select a treatment

- ◯ non-operative (includes medications, injections, physical therapy, or watchful waiting)
- ◯ arthroscopic treatment
- ◯ hemiarthroplasty
- ◯ reverse shoulder arthroplasty

Case 17

A 45 y.o. who works a desk job and enjoys playing rec basketball and lifting weights presents with severe signs and symptoms (constant)

* 49. Please grade the radiographs according to Seebauer

- ◯ **Type 1A-Centered, Stable:** minimal superior migration, C-A arch acetabularization and femoralization of humeral head
- ◯ **Type 1B-Centered, Medialized:** minimal superior migration, medial glenoid erosion, C-A arch acetabularization and femoralization of humeral head
- ◯ **Type 2A-Decentered, Limited Stable:** superior migration, superior-medial erosion, extensive C-A arch acetabularization and femoralization of humeral head
- ◯ **Type 2B-Decentered, Unstable:** anterior-superior escape, absent stabilization, C-A arch and anterior structures deficient

* 50. Please grade the radiographs according to Hamada

- ◯ **Grade 1:** acromio-humeral interval (AHI) > 6mm
- ◯ **Grade 2:** acromio-humeral interval (AHI) 5mm or less
- ◯ **Grade 3:** grade 2 with acetabularization of acromion
- ◯ **Grade 4:** grade 3 with narrowing of gleno-humeral joint
- ◯ **Grade 5:** grade 4 with bony destruction and humeral head collapse

* 51. Please select a treatment

- ◯ non-operative (includes medications, injections, physical therapy, or watchful waiting)
- ◯ arthroscopic treatment
- ◯ hemiarthroplasty
- ◯ reverse shoulder arthroplasty

Case 18

An inactive 45 y.o. who is very low demand with their shoulder presents with severe signs and symptoms (constant)

* 52. Please grade the radiographs according to Seebauer

- **Type 1A-Centered, Stable:** minimal superior migration, C-A arch acetabularization and femoralization of humeral head
- **Type 1B-Centered, Medialized:** minimal superior migration, medial glenoid erosion, C-A arch acetabularization and femoralization of humeral head
- **Type 2A-Decentered, Limited Stable:** superior migration, superior-medial erosion, extensive C-A arch acetabularization and femoralization of humeral head
- **Type 2B-Decentered, Unstable:** anterior-superior escape, absent stabilization, C-A arch and anterior structures deficient

* 53. Please grade the radiographs according to Hamada

- **Grade 1:** acromio-humeral interval (AHI) > 6mm
- **Grade 2:** acromio-humeral interval (AHI) 5mm or less
- **Grade 3:** grade 2 with acetabularization of acromion
- **Grade 4:** grade 3 with narrowing of gleno-humeral joint
- **Grade 5:** grade 4 with bony destruction and humeral head collapse

* 54. Please select a treatment

- non-operative (includes medications, injections, physical therapy, or watchful waiting)
- arthroscopic treatment
- hemiarthroplasty
- reverse shoulder arthroplasty

Case 19

An active 65 y.o. who works as a manual laborer presents with mild signs and symptoms (only during strenuous activities)

* 55. Please grade the radiographs according to Seebauer

- ◯ **Type 1A-Centered, Stable:** minimal superior migration, C-A arch acetabularization and femoralization of humeral head
- ◯ **Type 1B-Centered, Medialized:** minimal superior migration, medial glenoid erosion, C-A arch acetabularization and femoralization of humeral head
- ◯ **Type 2A-Decentered, Limited Stable:** superior migration, superior-medial erosion, extensive C-A arch acetabularization and femoralization of humeral head
- ◯ **Type 2B-Decentered, Unstable:** anterior-superior escape, absent stabilization, C-A arch and anterior structures deficient

* 56. Please grade the radiographs according to Hamada

- ◯ **Grade 1:** acromio-humeral interval (AHI) > 6mm
- ◯ **Grade 2:** acromio-humeral interval (AHI) 5mm or less
- ◯ **Grade 3:** grade 2 with acetabularization of acromion
- ◯ **Grade 4:** grade 3 with narrowing of gleno-humeral joint
- ◯ **Grade 5:** grade 4 with bony destruction and humeral head collapse

* 57. Please select a treatment

- ◯ non-operative (includes medications, injections, physical therapy, or watchful waiting)
- ◯ arthroscopic treatment
- ◯ hemiarthroplasty
- ◯ reverse shoulder arthroplasty

Case 20

A 65 y.o. who works a desk job and enjoys playing rec basketball and lifting weights presents with mild signs and symptoms (only during strenuous activities)

* 58. Please grade the radiographs according to Seebauer

- ◯ **Type 1A-Centered, Stable:** minimal superior migration, C-A arch acetabularization and femoralization of humeral head
- ◯ **Type 1B-Centered, Medialized:** minimal superior migration, medial glenoid erosion, C-A arch acetabularization and femoralization of humeral head
- ◯ **Type 2A-Decentered, Limited Stable:** superior migration, superior-medial erosion, extensive C-A arch acetabularization and femoralization of humeral head
- ◯ **Type 2B-Decentered, Unstable:** anterior-superior escape, absent stabilization, C-A arch and anterior structures deficient

* 59. Please grade the radiographs according to Hamada

- ◯ **Grade 1:** acromio-humeral interval (AHI) > 6mm
- ◯ **Grade 2:** acromio-humeral interval (AHI) 5mm or less
- ◯ **Grade 3:** grade 2 with acetabularization of acromion
- ◯ **Grade 4:** grade 3 with narrowing of gleno-humeral joint
- ◯ **Grade 5:** grade 4 with bony destruction and humeral head collapse

* 60. Please select a treatment

- ◯ non-operative (includes medications, injections, physical therapy, or watchful waiting)
- ◯ arthroscopic treatment
- ◯ hemiarthroplasty
- ◯ reverse shoulder arthroplasty

Case 21

An inactive 65 y.o. who is very low demand with their shoulder presents with mild signs and symptoms (only during strenuous activities)

*** 61. Please grade the radiographs according to Seebauer**

- ○ **Type 1A-Centered, Stable:** minimal superior migration, C-A arch acetabularization and femoralization of humeral head
- ○ **Type 1B-Centered, Medialized:** minimal superior migration, medial glenoid erosion, C-A arch acetabularization and femoralization of humeral head
- ○ **Type 2A-Decentered, Limited Stable:** superior migration, superior-medial erosion, extensive C-A arch acetabularization and femoralization of humeral head
- ○ **Type 2B-Decentered, Unstable:** anterior-superior escape, absent stabilization, C-A arch and anterior structures deficient

*** 62. Please grade the radiographs according to Hamada**

- ○ **Grade 1:** acromio-humeral interval (AHI) > 6mm
- ○ **Grade 2:** acromio-humeral interval (AHI) 5mm or less
- ○ **Grade 3:** grade 2 with acetabularization of acromion
- ○ **Grade 4:** grade 3 with narrowing of gleno-humeral joint
- ○ **Grade 5:** grade 4 with bony destruction and humeral head collapse

*** 63. Please select a treatment**

- ○ non-operative (includes medications, injections, physical therapy, or watchful waiting)
- ○ arthroscopic treatment
- ○ hemiarthroplasty
- ○ reverse shoulder arthroplasty

Case 22

An active 65 y.o. who works as a manual laborer presents with moderate signs and symptoms (predominant symptoms that limit most things)

*** 64. Please grade the radiographs according to Seebauer**

- ◯ **Type 1A-Centered, Stable:** minimal superior migration, C-A arch acetabularization and femoralization of humeral head
- ◯ **Type 1B-Centered, Medialized:** minimal superior migration, medial glenoid erosion, C-A arch acetabularization and femoralization of humeral head
- ◯ **Type 2A-Decentered, Limited Stable:** superior migration, superior-medial erosion, extensive C-A arch acetabularization and femoralization of humeral head
- ◯ **Type 2B-Decentered, Unstable:** anterior-superior escape, absent stabilization, C-A arch and anterior structures deficient

*** 65. Please grade the radiographs according to Hamada**

- ◯ **Grade 1:** acromio-humeral interval (AHI) > 6mm
- ◯ **Grade 2:** acromio-humeral interval (AHI) 5mm or less
- ◯ **Grade 3:** grade 2 with acetabularization of acromion
- ◯ **Grade 4:** grade 3 with narrowing of gleno-humeral joint
- ◯ **Grade 5:** grade 4 with bony destruction and humeral head collapse

*** 66. Please select a treatment**

- ◯ non-operative (includes medications, injections, physical therapy, or watchful waiting)
- ◯ arthroscopic treatment
- ◯ hemiarthroplasty
- ◯ reverse shoulder arthroplasty

Case 23

A 65 y.o. who works a desk job and enjoys playing rec basketball and lifting weights presents with moderate signs and symptoms (predominant symptoms that limit most things)

* 67. Please grade the radiographs according to Seebauer

- ◯ **Type 1A-Centered, Stable:** minimal superior migration, C-A arch acetabularization and femoralization of humeral head
- ◯ **Type 1B-Centered, Medialized:** minimal superior migration, medial glenoid erosion, C-A arch acetabularization and femoralization of humeral head
- ◯ **Type 2A-Decentered, Limited Stable:** superior migration, superior-medial erosion, extensive C-A arch acetabularization and femoralization of humeral head
- ◯ **Type 2B-Decentered, Unstable:** anterior-superior escape, absent stabilization, C-A arch and anterior structures deficient

* 68. Please grade the radiographs according to Hamada

- ◯ **Grade 1:** acromio-humeral interval (AHI) > 6mm
- ◯ **Grade 2:** acromio-humeral interval (AHI) 5mm or less
- ◯ **Grade 3:** grade 2 with acetabularization of acromion
- ◯ **Grade 4:** grade 3 with narrowing of gleno-humeral joint
- ◯ **Grade 5:** grade 4 with bony destruction and humeral head collapse

* 69. Please select a treatment

- ◯ non-operative (includes medications, injections, physical therapy, or watchful waiting)
- ◯ arthroscopic treatment
- ◯ hemiarthroplasty
- ◯ reverse shoulder arthroplasty

Case 24

An inactive 65 y.o. who is very low demand with their shoulder presents with moderate signs and symptoms (predominant symptoms that limit most things)

* 70. Please grade the radiographs according to Seebauer

- ◯ **Type 1A-Centered, Stable:** minimal superior migration, C-A arch acetabularization and femoralization of humeral head
- ◯ **Type 1B-Centered, Medialized:** minimal superior migration, medial glenoid erosion, C-A arch acetabularization and femoralization of humeral head
- ◯ **Type 2A-Decentered, Limited Stable:** superior migration, superior-medial erosion, extensive C-A arch acetabularization and femoralization of humeral head
- ◯ **Type 2B-Decentered, Unstable:** anterior-superior escape, absent stabilization, C-A arch and anterior structures deficient

* 71. Please grade the radiographs according to Hamada

- ◯ **Grade 1:** acromio-humeral interval (AHI) > 6mm
- ◯ **Grade 2:** acromio-humeral interval (AHI) 5mm or less
- ◯ **Grade 3:** grade 2 with acetabularization of acromion
- ◯ **Grade 4:** grade 3 with narrowing of gleno-humeral joint
- ◯ **Grade 5:** grade 4 with bony destruction and humeral head collapse

* 72. Please select a treatment

- ◯ non-operative (includes medications, injections, physical therapy, or watchful waiting)
- ◯ arthroscopic treatment
- ◯ hemiarthroplasty
- ◯ reverse shoulder arthroplasty

Case 25

An active 65 y.o. who works as a manual laborer presents with severe signs and symptoms (constant)

* 73. Please grade the radiographs according to Seebauer

- ◯ **Type 1A-Centered, Stable:** minimal superior migration, C-A arch acetabularization and femoralization of humeral head
- ◯ **Type 1B-Centered, Medialized:** minimal superior migration, medial glenoid erosion, C-A arch acetabularization and femoralization of humeral head
- ◯ **Type 2A-Decentered, Limited Stable:** superior migration, superior-medial erosion, extensive C-A arch acetabularization and femoralization of humeral head
- ◯ **Type 2B-Decentered, Unstable:** anterior-superior escape, absent stabilization, C-A arch and anterior structures deficient

* 74. Please grade the radiographs according to Hamada

- ◯ **Grade 1:** acromio-humeral interval (AHI) > 6mm
- ◯ **Grade 2:** acromio-humeral interval (AHI) 5mm or less
- ◯ **Grade 3:** grade 2 with acetabularization of acromion
- ◯ **Grade 4:** grade 3 with narrowing of gleno-humeral joint
- ◯ **Grade 5:** grade 4 with bony destruction and humeral head collapse

* 75. Please select a treatment

- ◯ non-operative (includes medications, injections, physical therapy, or watchful waiting)
- ◯ arthroscopic treatment
- ◯ hemiarthroplasty
- ◯ reverse shoulder arthroplasty

Case 26

A 65 y.o. who works a desk job and enjoys playing rec basketball and lifting weights presents with severe signs and symptoms (constant)

*** 76. Please grade the radiographs according to Seebauer**

- **Type 1A-Centered, Stable:** minimal superior migration, C-A arch acetabularization and femoralization of humeral head
- **Type 1B-Centered, Medialized:** minimal superior migration, medial glenoid erosion, C-A arch acetabularization and femoralization of humeral head
- **Type 2A-Decentered, Limited Stable:** superior migration, superior-medial erosion, extensive C-A arch acetabularization and femoralization of humeral head
- **Type 2B-Decentered, Unstable:** anterior-superior escape, absent stabilization, C-A arch and anterior structures deficient

*** 77. Please grade the radiographs according to Hamada**

- **Grade 1:** acromio-humeral interval (AHI) > 6mm
- **Grade 2:** acromio-humeral interval (AHI) 5mm or less
- **Grade 3:** grade 2 with acetabularization of acromion
- **Grade 4:** grade 3 with narrowing of gleno-humeral joint
- **Grade 5:** grade 4 with bony destruction and humeral head collapse

*** 78. Please select a treatment**

- non-operative (includes medications, injections, physical therapy, or watchful waiting)
- arthroscopic treatment
- hemiarthroplasty
- reverse shoulder arthroplasty

Case 27

An inactive 65 y.o. who is very low demand with their shoulder presents with severe signs and symptoms (constant)

* 79. Please grade the radiographs according to Seebauer

- ◯ **Type 1A-Centered, Stable:** minimal superior migration, C-A arch acetabularization and femoralization of humeral head
- ◯ **Type 1B-Centered, Medialized:** minimal superior migration, medial glenoid erosion, C-A arch acetabularization and femoralization of humeral head
- ◯ **Type 2A-Decentered, Limited Stable:** superior migration, superior-medial erosion, extensive C-A arch acetabularization and femoralization of humeral head
- ◯ **Type 2B-Decentered, Unstable:** anterior-superior escape, absent stabilization, C-A arch and anterior structures deficient

* 80. Please grade the radiographs according to Hamada

- ◯ **Grade 1:** acromio-humeral interval (AHI) > 6mm
- ◯ **Grade 2:** acromio-humeral interval (AHI) 5mm or less
- ◯ **Grade 3:** grade 2 with acetabularization of acromion
- ◯ **Grade 4:** grade 3 with narrowing of gleno-humeral joint
- ◯ **Grade 5:** grade 4 with bony destruction and humeral head collapse

* 81. Please select a treatment

- ◯ non-operative (includes medications, injections, physical therapy, or watchful waiting)
- ◯ arthroscopic treatment
- ◯ hemiarthroplasty
- ◯ reverse shoulder arthroplasty

Case 28

An active 30 y.o. who works as a manual laborer presents with mild signs and symptoms (only during strenuous activities)

* 82. Please grade the radiographs according to Seebauer

- ◯ **Type 1A-Centered, Stable:** minimal superior migration, C-A arch acetabularization and femoralization of humeral head
- ◯ **Type 1B-Centered, Medialized:** minimal superior migration, medial glenoid erosion, C-A arch acetabularization and femoralization of humeral head
- ◯ **Type 2A-Decentered, Limited Stable:** superior migration, superior-medial erosion, extensive C-A arch acetabularization and femoralization of humeral head
- ◯ **Type 2B-Decentered, Unstable:** anterior-superior escape, absent stabilization, C-A arch and anterior structures deficient

* 83. Please grade the radiographs according to Hamada

- ◯ **Grade 1:** acromio-humeral interval (AHI) > 6mm
- ◯ **Grade 2:** acromio-humeral interval (AHI) 5mm or less
- ◯ **Grade 3:** grade 2 with acetabularization of acromion
- ◯ **Grade 4:** grade 3 with narrowing of gleno-humeral joint
- ◯ **Grade 5:** grade 4 with bony destruction and humeral head collapse

* 84. Please select a treatment

- ◯ non-operative (includes medications, injections, physical therapy, or watchful waiting)
- ◯ arthroscopic treatment
- ◯ hemiarthroplasty
- ◯ reverse shoulder arthroplasty

Case 29

A 30 y.o. who works a desk job and enjoys playing rec basketball and lifting weights presents with mild signs and symptoms (only during strenuous activities)

*** 85. Please grade the radiographs according to Seebauer**

- **Type 1A-Centered, Stable:** minimal superior migration, C-A arch acetabularization and femoralization of humeral head
- **Type 1B-Centered, Medialized:** minimal superior migration, medial glenoid erosion, C-A arch acetabularization and femoralization of humeral head
- **Type 2A-Decentered, Limited Stable:** superior migration, superior-medial erosion, extensive C-A arch acetabularization and femoralization of humeral head
- **Type 2B-Decentered, Unstable:** anterior-superior escape, absent stabilization, C-A arch and anterior structures deficient

*** 86. Please grade the radiographs according to Hamada**

- **Grade 1:** acromio-humeral interval (AHI) > 6mm
- **Grade 2:** acromio-humeral interval (AHI) 5mm or less
- **Grade 3:** grade 2 with acetabularization of acromion
- **Grade 4:** grade 3 with narrowing of gleno-humeral joint
- **Grade 5:** grade 4 with bony destruction and humeral head collapse

*** 87. Please select a treatment**

- non-operative (includes medications, injections, physical therapy, or watchful waiting)
- arthroscopic treatment
- hemiarthroplasty
- reverse shoulder arthroplasty

Case 30

An inactive 30 y.o. who is very low demand with their shoulder presents with mild signs and symptoms (only during strenuous activities)

* 88. Please grade the radiographs according to Seebauer

- **Type 1A-Centered, Stable:** minimal superior migration, C-A arch acetabularization and femoralization of humeral head
- **Type 1B-Centered, Medialized:** minimal superior migration, medial glenoid erosion, C-A arch acetabularization and femoralization of humeral head
- **Type 2A-Decentered, Limited Stable:** superior migration, superior-medial erosion, extensive C-A arch acetabularization and femoralization of humeral head
- **Type 2B-Decentered, Unstable:** anterior-superior escape, absent stabilization, C-A arch and anterior structures deficient

* 89. Please grade the radiographs according to Hamada

- **Grade 1:** acromio-humeral interval (AHI) > 6mm
- **Grade 2:** acromio-humeral interval (AHI) 5mm or less
- **Grade 3:** grade 2 with acetabularization of acromion
- **Grade 4:** grade 3 with narrowing of gleno-humeral joint
- **Grade 5:** grade 4 with bony destruction and humeral head collapse

* 90. Please select a treatment

- non-operative (includes medications, injections, physical therapy, or watchful waiting)
- arthroscopic treatment
- hemiarthroplasty
- reverse shoulder arthroplasty

Case 31

An active 30 y.o. who works as a manual laborer presents with moderate signs and symptoms (predominant symptoms that limit most things)

* 91. Please grade the radiographs according to Seebauer

- ◯ **Type 1A-Centered, Stable:** minimal superior migration, C-A arch acetabularization and femoralization of humeral head
- ◯ **Type 1B-Centered, Medialized:** minimal superior migration, medial glenoid erosion, C-A arch acetabularization and femoralization of humeral head
- ◯ **Type 2A-Decentered, Limited Stable:** superior migration, superior-medial erosion, extensive C-A arch acetabularization and femoralization of humeral head
- ◯ **Type 2B-Decentered, Unstable:** anterior-superior escape, absent stabilization, C-A arch and anterior structures deficient

* 92. Please grade the radiographs according to Hamada

- ◯ **Grade 1:** acromio-humeral interval (AHI) > 6mm
- ◯ **Grade 2:** acromio-humeral interval (AHI) 5mm or less
- ◯ **Grade 3:** grade 2 with acetabularization of acromion
- ◯ **Grade 4:** grade 3 with narrowing of gleno-humeral joint
- ◯ **Grade 5:** grade 4 with bony destruction and humeral head collapse

* 93. Please select a treatment

- ◯ non-operative (includes medications, injections, physical therapy, or watchful waiting)
- ◯ arthroscopic treatment
- ◯ hemiarthroplasty
- ◯ reverse shoulder arthroplasty

Case 32

A 30 y.o. who works a desk job and enjoys playing rec basketball and lifting weights presents with moderate signs and symptoms (predominant symptoms that limit most things)

* 94. Please grade the radiographs according to Seebauer

- ◯ **Type 1A-Centered, Stable:** minimal superior migration, C-A arch acetabularization and femoralization of humeral head
- ◯ **Type 1B-Centered, Medialized:** minimal superior migration, medial glenoid erosion, C-A arch acetabularization and femoralization of humeral head
- ◯ **Type 2A-Decentered, Limited Stable:** superior migration, superior-medial erosion, extensive C-A arch acetabularization and femoralization of humeral head
- ◯ **Type 2B-Decentered, Unstable:** anterior-superior escape, absent stabilization, C-A arch and anterior structures deficient

* 95. Please grade the radiographs according to Hamada

- ◯ **Grade 1:** acromio-humeral interval (AHI) > 6mm
- ◯ **Grade 2:** acromio-humeral interval (AHI) 5mm or less
- ◯ **Grade 3:** grade 2 with acetabularization of acromion
- ◯ **Grade 4:** grade 3 with narrowing of gleno-humeral joint
- ◯ **Grade 5:** grade 4 with bony destruction and humeral head collapse

* 96. Please select a treatment

- ◯ non-operative (includes medications, injections, physical therapy, or watchful waiting)
- ◯ arthroscopic treatment
- ◯ hemiarthroplasty
- ◯ reverse shoulder arthroplasty

Case 33

An inactive 30 y.o. who is very low demand with their shoulder presents with moderate signs and symptoms (predominant symptoms that limit most things)

* 97. Please grade the radiographs according to Seebauer

- ◯ **Type 1A-Centered, Stable:** minimal superior migration, C-A arch acetabularization and femoralization of humeral head
- ◯ **Type 1B-Centered, Medialized:** minimal superior migration, medial glenoid erosion, C-A arch acetabularization and femoralization of humeral head
- ◯ **Type 2A-Decentered, Limited Stable:** superior migration, superior-medial erosion, extensive C-A arch acetabularization and femoralization of humeral head
- ◯ **Type 2B-Decentered, Unstable:** anterior-superior escape, absent stabilization, C-A arch and anterior structures deficient

* 98. Please grade the radiographs according to Hamada

- ◯ **Grade 1:** acromio-humeral interval (AHI) > 6mm
- ◯ **Grade 2:** acromio-humeral interval (AHI) 5mm or less
- ◯ **Grade 3:** grade 2 with acetabularization of acromion
- ◯ **Grade 4:** grade 3 with narrowing of gleno-humeral joint
- ◯ **Grade 5:** grade 4 with bony destruction and humeral head collapse

* 99. Please select a treatment

- ◯ non-operative (includes medications, injections, physical therapy, or watchful waiting)
- ◯ arthroscopic treatment
- ◯ hemiarthroplasty
- ◯ reverse shoulder arthroplasty

Case 34

An active 30 y.o. who works as a manual laborer presents with severe signs and symptoms (constant)

* 100. Please grade the radiographs according to Seebauer

- ◯ **Type 1A-Centered, Stable:** minimal superior migration, C-A arch acetabularization and femoralization of humeral head
- ◯ **Type 1B-Centered, Medialized:** minimal superior migration, medial glenoid erosion, C-A arch acetabularization and femoralization of humeral head
- ◯ **Type 2A-Decentered, Limited Stable:** superior migration, superior-medial erosion, extensive C-A arch acetabularization and femoralization of humeral head
- ◯ **Type 2B-Decentered, Unstable:** anterior-superior escape, absent stabilization, C-A arch and anterior structures deficient

* 101. Please grade the radiographs according to Hamada

- ◯ **Grade 1:** acromio-humeral interval (AHI) > 6mm
- ◯ **Grade 2:** acromio-humeral interval (AHI) 5mm or less
- ◯ **Grade 3:** grade 2 with acetabularization of acromion
- ◯ **Grade 4:** grade 3 with narrowing of gleno-humeral joint
- ◯ **Grade 5:** grade 4 with bony destruction and humeral head collapse

* 102. Please select a treatment

- ◯ non-operative (includes medications, injections, physical therapy, or watchful waiting)
- ◯ arthroscopic treatment
- ◯ hemiarthroplasty
- ◯ reverse shoulder arthroplasty

Case 35

A 30 y.o. who works a desk job and enjoys playing rec basketball and lifting weights presents with severe signs and symptoms (constant)

* 103. Please grade the radiographs according to Seebauer

- ◯ **Type 1A-Centered, Stable:** minimal superior migration, C-A arch acetabularization and femoralization of humeral head
- ◯ **Type 1B-Centered, Medialized:** minimal superior migration, medial glenoid erosion, C-A arch acetabularization and femoralization of humeral head
- ◯ **Type 2A-Decentered, Limited Stable:** superior migration, superior-medial erosion, extensive C-A arch acetabularization and femoralization of humeral head
- ◯ **Type 2B-Decentered, Unstable:** anterior-superior escape, absent stabilization, C-A arch and anterior structures deficient

* 104. Please grade the radiographs according to Hamada

- ◯ **Grade 1:** acromio-humeral interval (AHI) > 6mm
- ◯ **Grade 2:** acromio-humeral interval (AHI) 5mm or less
- ◯ **Grade 3:** grade 2 with acetabularization of acromion
- ◯ **Grade 4:** grade 3 with narrowing of gleno-humeral joint
- ◯ **Grade 5:** grade 4 with bony destruction and humeral head collapse

* 105. Please select a treatment

- ◯ non-operative (includes medications, injections, physical therapy, or watchful waiting)
- ◯ arthroscopic treatment
- ◯ hemiarthroplasty
- ◯ reverse shoulder arthroplasty

Case 36

An inactive 30 y.o. who is very low demand with their shoulder presents with severe signs and symptoms (constant)

* 106. Please grade the radiographs according to Seebauer

- **Type 1A-Centered, Stable:** minimal superior migration, C-A arch acetabularization and femoralization of humeral head
- **Type 1B-Centered, Medialized:** minimal superior migration, medial glenoid erosion, C-A arch acetabularization and femoralization of humeral head
- **Type 2A-Decentered, Limited Stable:** superior migration, superior-medial erosion, extensive C-A arch acetabularization and femoralization of humeral head
- **Type 2B-Decentered, Unstable:** anterior-superior escape, absent stabilization, C-A arch and anterior structures deficient

* 107. Please grade the radiographs according to Hamada

- **Grade 1:** acromio-humeral interval (AHI) > 6mm
- **Grade 2:** acromio-humeral interval (AHI) 5mm or less
- **Grade 3:** grade 2 with acetabularization of acromion
- **Grade 4:** grade 3 with narrowing of gleno-humeral joint
- **Grade 5:** grade 4 with bony destruction and humeral head collapse

* 108. Please select a treatment

- non-operative (includes medications, injections, physical therapy, or watchful waiting)
- arthroscopic treatment
- hemiarthroplasty
- reverse shoulder arthroplasty

Case 37

An active 45 y.o. who works as a manual laborer presents with mild signs and symptoms (only during strenuous activities)

* 109. Please grade the radiographs according to Seebauer

- ◯ **Type 1A-Centered, Stable:** minimal superior migration, C-A arch acetabularization and femoralization of humeral head
- ◯ **Type 1B-Centered, Medialized:** minimal superior migration, medial glenoid erosion, C-A arch acetabularization and femoralization of humeral head
- ◯ **Type 2A-Decentered, Limited Stable:** superior migration, superior-medial erosion, extensive C-A arch acetabularization and femoralization of humeral head
- ◯ **Type 2B-Decentered, Unstable:** anterior-superior escape, absent stabilization, C-A arch and anterior structures deficient

* 110. Please grade the radiographs according to Hamada

- ◯ **Grade 1:** acromio-humeral interval (AHI) > 6mm
- ◯ **Grade 2:** acromio-humeral interval (AHI) 5mm or less
- ◯ **Grade 3:** grade 2 with acetabularization of acromion
- ◯ **Grade 4:** grade 3 with narrowing of gleno-humeral joint
- ◯ **Grade 5:** grade 4 with bony destruction and humeral head collapse

* 111. Please select a treatment

- ◯ non-operative (includes medications, injections, physical therapy, or watchful waiting)
- ◯ arthroscopic treatment
- ◯ hemiarthroplasty
- ◯ reverse shoulder arthroplasty

Case 38

A 45 y.o. who works a desk job and enjoys playing rec basketball and lifting weights presents with mild signs and symptoms (only during strenuous activities)

* 112. Please grade the radiographs according to Seebauer

- ◯ **Type 1A-Centered, Stable:** minimal superior migration, C-A arch acetabularization and femoralization of humeral head
- ◯ **Type 1B-Centered, Medialized:** minimal superior migration, medial glenoid erosion, C-A arch acetabularization and femoralization of humeral head
- ◯ **Type 2A-Decentered, Limited Stable:** superior migration, superior-medial erosion, extensive C-A arch acetabularization and femoralization of humeral head
- ◯ **Type 2B-Decentered, Unstable:** anterior-superior escape, absent stabilization, C-A arch and anterior structures deficient

* 113. Please grade the radiographs according to Hamada

- ◯ **Grade 1:** acromio-humeral interval (AHI) > 6mm
- ◯ **Grade 2:** acromio-humeral interval (AHI) 5mm or less
- ◯ **Grade 3:** grade 2 with acetabularization of acromion
- ◯ **Grade 4:** grade 3 with narrowing of gleno-humeral joint
- ◯ **Grade 5:** grade 4 with bony destruction and humeral head collapse

* 114. Please select a treatment

- ◯ non-operative (includes medications, injections, physical therapy, or watchful waiting)
- ◯ arthroscopic treatment
- ◯ hemiarthroplasty
- ◯ reverse shoulder arthroplasty

Case 39

An inactive 45 y.o. who is very low demand with their shoulder presents with mild signs and symptoms (only during strenuous activities)

* 115. Please grade the radiographs according to Seebauer

- **Type 1A-Centered, Stable:** minimal superior migration, C-A arch acetabularization and femoralization of humeral head
- **Type 1B-Centered, Medialized:** minimal superior migration, medial glenoid erosion, C-A arch acetabularization and femoralization of humeral head
- **Type 2A-Decentered, Limited Stable:** superior migration, superior-medial erosion, extensive C-A arch acetabularization and femoralization of humeral head
- **Type 2B-Decentered, Unstable:** anterior-superior escape, absent stabilization, C-A arch and anterior structures deficient

* 116. Please grade the radiographs according to Hamada

- **Grade 1:** acromio-humeral interval (AHI) > 6mm
- **Grade 2:** acromio-humeral interval (AHI) 5mm or less
- **Grade 3:** grade 2 with acetabularization of acromion
- **Grade 4:** grade 3 with narrowing of gleno-humeral joint
- **Grade 5:** grade 4 with bony destruction and humeral head collapse

* 117. Please select a treatment

- non-operative (includes medications, injections, physical therapy, or watchful waiting)
- arthroscopic treatment
- hemiarthroplasty
- reverse shoulder arthroplasty

Case 40

An active 45 y.o. who works as a manual laborer presents with moderate signs and symptoms (predominant symptoms that limit most things)

* 118. Please grade the radiographs according to Seebauer

- ◯ **Type 1A-Centered, Stable:** minimal superior migration, C-A arch acetabularization and femoralization of humeral head
- ◯ **Type 1B-Centered, Medialized:** minimal superior migration, medial glenoid erosion, C-A arch acetabularization and femoralization of humeral head
- ◯ **Type 2A-Decentered, Limited Stable:** superior migration, superior-medial erosion, extensive C-A arch acetabularization and femoralization of humeral head
- ◯ **Type 2B-Decentered, Unstable:** anterior-superior escape, absent stabilization, C-A arch and anterior structures deficient

* 119. Please grade the radiographs according to Hamada

- ◯ **Grade 1:** acromio-humeral interval (AHI) > 6mm
- ◯ **Grade 2:** acromio-humeral interval (AHI) 5mm or less
- ◯ **Grade 3:** grade 2 with acetabularization of acromion
- ◯ **Grade 4:** grade 3 with narrowing of gleno-humeral joint
- ◯ **Grade 5:** grade 4 with bony destruction and humeral head collapse

* 120. Please select a treatment

- ◯ non-operative (includes medications, injections, physical therapy, or watchful waiting)
- ◯ arthroscopic treatment
- ◯ hemiarthroplasty
- ◯ reverse shoulder arthroplasty

Case 41

A 45 y.o. who works a desk job and enjoys playing rec basketball and lifting weights presents with moderate signs and symptoms (predominant symptoms that limit most things)

* 121. Please grade the radiographs according to Seebauer

- ◯ **Type 1A-Centered, Stable:** minimal superior migration, C-A arch acetabularization and femoralization of humeral head
- ◯ **Type 1B-Centered, Medialized:** minimal superior migration, medial glenoid erosion, C-A arch acetabularization and femoralization of humeral head
- ◯ **Type 2A-Decentered, Limited Stable:** superior migration, superior-medial erosion, extensive C-A arch acetabularization and femoralization of humeral head
- ◯ **Type 2B-Decentered, Unstable:** anterior-superior escape, absent stabilization, C-A arch and anterior structures deficient

* 122. Please grade the radiographs according to Hamada

- ◯ **Grade 1:** acromio-humeral interval (AHI) > 6mm
- ◯ **Grade 2:** acromio-humeral interval (AHI) 5mm or less
- ◯ **Grade 3:** grade 2 with acetabularization of acromion
- ◯ **Grade 4:** grade 3 with narrowing of gleno-humeral joint
- ◯ **Grade 5:** grade 4 with bony destruction and humeral head collapse

* 123. Please select a treatment

- ◯ non-operative (includes medications, injections, physical therapy, or watchful waiting)
- ◯ arthroscopic treatment
- ◯ hemiarthroplasty
- ◯ reverse shoulder arthroplasty

Case 42

An inactive 45 y.o. who is very low demand with their shoulder presents with moderate signs and symptoms (predominant symptoms that limit most things)

* 124. Please grade the radiographs according to Seebauer

- ○ **Type 1A-Centered, Stable:** minimal superior migration, C-A arch acetabularization and femoralization of humeral head
- ○ **Type 1B-Centered, Medialized:** minimal superior migration, medial glenoid erosion, C-A arch acetabularization and femoralization of humeral head
- ○ **Type 2A-Decentered, Limited Stable:** superior migration, superior-medial erosion, extensive C-A arch acetabularization and femoralization of humeral head
- ○ **Type 2B-Decentered, Unstable:** anterior-superior escape, absent stabilization, C-A arch and anterior structures deficient

* 125. Please grade the radiographs according to Hamada

- ○ **Grade 1:** acromio-humeral interval (AHI) > 6mm
- ○ **Grade 2:** acromio-humeral interval (AHI) 5mm or less
- ○ **Grade 3:** grade 2 with acetabularization of acromion
- ○ **Grade 4:** grade 3 with narrowing of gleno-humeral joint
- ○ **Grade 5:** grade 4 with bony destruction and humeral head collapse

* 126. Please select a treatment

- ○ non-operative (includes medications, injections, physical therapy, or watchful waiting)
- ○ arthroscopic treatment
- ○ hemiarthroplasty
- ○ reverse shoulder arthroplasty

Case 43

An active 45 y.o. who works as a manual laborer presents with severe signs and symptoms (constant)

*** 127. Please grade the radiographs according to Seebauer**

- ◯ **Type 1A-Centered, Stable:** minimal superior migration, C-A arch acetabularization and femoralization of humeral head
- ◯ **Type 1B-Centered, Medialized:** minimal superior migration, medial glenoid erosion, C-A arch acetabularization and femoralization of humeral head
- ◯ **Type 2A-Decentered, Limited Stable:** superior migration, superior-medial erosion, extensive C-A arch acetabularization and femoralization of humeral head
- ◯ **Type 2B-Decentered, Unstable:** anterior-superior escape, absent stabilization, C-A arch and anterior structures deficient

*** 128. Please grade the radiographs according to Hamada**

- ◯ **Grade 1:** acromio-humeral interval (AHI) > 6mm
- ◯ **Grade 2:** acromio-humeral interval (AHI) 5mm or less
- ◯ **Grade 3:** grade 2 with acetabularization of acromion
- ◯ **Grade 4:** grade 3 with narrowing of gleno-humeral joint
- ◯ **Grade 5:** grade 4 with bony destruction and humeral head collapse

*** 129. Please select a treatment**

- ◯ non-operative (includes medications, injections, physical therapy, or watchful waiting)
- ◯ arthroscopic treatment
- ◯ hemiarthroplasty
- ◯ reverse shoulder arthroplasty

Case 44

A 45 y.o. who works a desk job and enjoys playing rec basketball and lifting weights presents with severe signs and symptoms (constant)

* 130. Please grade the radiographs according to Seebauer

- ◯ **Type 1A-Centered, Stable:** minimal superior migration, C-A arch acetabularization and femoralization of humeral head
- ◯ **Type 1B-Centered, Medialized:** minimal superior migration, medial glenoid erosion, C-A arch acetabularization and femoralization of humeral head
- ◯ **Type 2A-Decentered, Limited Stable:** superior migration, superior-medial erosion, extensive C-A arch acetabularization and femoralization of humeral head
- ◯ **Type 2B-Decentered, Unstable:** anterior-superior escape, absent stabilization, C-A arch and anterior structures deficient

* 131. Please grade the radiographs according to Hamada

- ◯ **Grade 1:** acromio-humeral interval (AHI) > 6mm
- ◯ **Grade 2:** acromio-humeral interval (AHI) 5mm or less
- ◯ **Grade 3:** grade 2 with acetabularization of acromion
- ◯ **Grade 4:** grade 3 with narrowing of gleno-humeral joint
- ◯ **Grade 5:** grade 4 with bony destruction and humeral head collapse

* 132. Please select a treatment

- ◯ non-operative (includes medications, injections, physical therapy, or watchful waiting)
- ◯ arthroscopic treatment
- ◯ hemiarthroplasty
- ◯ reverse shoulder arthroplasty

Case 45

An inactive 45 y.o. who is very low demand with their shoulder presents with severe signs and symptoms (constant)

* 133. Please grade the radiographs according to Seebauer

- ◯ **Type 1A-Centered, Stable:** minimal superior migration, C-A arch acetabularization and femoralization of humeral head
- ◯ **Type 1B-Centered, Medialized:** minimal superior migration, medial glenoid erosion, C-A arch acetabularization and femoralization of humeral head
- ◯ **Type 2A-Decentered, Limited Stable:** superior migration, superior-medial erosion, extensive C-A arch acetabularization and femoralization of humeral head
- ◯ **Type 2B-Decentered, Unstable:** anterior-superior escape, absent stabilization, C-A arch and anterior structures deficient

* 134. Please grade the radiographs according to Hamada

- ◯ **Grade 1:** acromio-humeral interval (AHI) > 6mm
- ◯ **Grade 2:** acromio-humeral interval (AHI) 5mm or less
- ◯ **Grade 3:** grade 2 with acetabularization of acromion
- ◯ **Grade 4:** grade 3 with narrowing of gleno-humeral joint
- ◯ **Grade 5:** grade 4 with bony destruction and humeral head collapse

* 135. Please select a treatment

- ◯ non-operative (includes medications, injections, physical therapy, or watchful waiting)
- ◯ arthroscopic treatment
- ◯ hemiarthroplasty
- ◯ reverse shoulder arthroplasty

Case 46

An active 65 y.o. who works as a manual laborer presents with mild signs and symptoms (only during strenuous activities)

* 136. Please grade the radiographs according to Seebauer

- ◯ **Type 1A-Centered, Stable:** minimal superior migration, C-A arch acetabularization and femoralization of humeral head
- ◯ **Type 1B-Centered, Medialized:** minimal superior migration, medial glenoid erosion, C-A arch acetabularization and femoralization of humeral head
- ◯ **Type 2A-Decentered, Limited Stable:** superior migration, superior-medial erosion, extensive C-A arch acetabularization and femoralization of humeral head
- ◯ **Type 2B-Decentered, Unstable:** anterior-superior escape, absent stabilization, C-A arch and anterior structures deficient

* 137. Please grade the radiographs according to Hamada

- ◯ **Grade 1:** acromio-humeral interval (AHI) > 6mm
- ◯ **Grade 2:** acromio-humeral interval (AHI) 5mm or less
- ◯ **Grade 3:** grade 2 with acetabularization of acromion
- ◯ **Grade 4:** grade 3 with narrowing of gleno-humeral joint
- ◯ **Grade 5:** grade 4 with bony destruction and humeral head collapse

* 138. Please select a treatment

- ◯ non-operative (includes medications, injections, physical therapy, or watchful waiting)
- ◯ arthroscopic treatment
- ◯ hemiarthroplasty
- ◯ reverse shoulder arthroplasty

Case 47

A 65 y.o. who works a desk job and enjoys playing rec basketball and lifting weights presents with mild signs and symptoms (only during strenuous activities)

* 139. Please grade the radiographs according to Seebauer

- **Type 1A-Centered, Stable:** minimal superior migration, C-A arch acetabularization and femoralization of humeral head
- **Type 1B-Centered, Medialized:** minimal superior migration, medial glenoid erosion, C-A arch acetabularization and femoralization of humeral head
- **Type 2A-Decentered, Limited Stable:** superior migration, superior-medial erosion, extensive C-A arch acetabularization and femoralization of humeral head
- **Type 2B-Decentered, Unstable:** anterior-superior escape, absent stabilization, C-A arch and anterior structures deficient

* 140. Please grade the radiographs according to Hamada

- **Grade 1:** acromio-humeral interval (AHI) > 6mm
- **Grade 2:** acromio-humeral interval (AHI) 5mm or less
- **Grade 3:** grade 2 with acetabularization of acromion
- **Grade 4:** grade 3 with narrowing of gleno-humeral joint
- **Grade 5:** grade 4 with bony destruction and humeral head collapse

* 141. Please select a treatment

- non-operative (includes medications, injections, physical therapy, or watchful waiting)
- arthroscopic treatment
- hemiarthroplasty
- reverse shoulder arthroplasty

Case 48

An inactive 65 y.o. who is very low demand with their shoulder presents with mild signs and symptoms (only during strenuous activities)

* 142. Please grade the radiographs according to Seebauer

- **Type 1A-Centered, Stable:** minimal superior migration, C-A arch acetabularization and femoralization of humeral head
- **Type 1B-Centered, Medialized:** minimal superior migration, medial glenoid erosion, C-A arch acetabularization and femoralization of humeral head
- **Type 2A-Decentered, Limited Stable:** superior migration, superior-medial erosion, extensive C-A arch acetabularization and femoralization of humeral head
- **Type 2B-Decentered, Unstable:** anterior-superior escape, absent stabilization, C-A arch and anterior structures deficient

* 143. Please grade the radiographs according to Hamada

- **Grade 1:** acromio-humeral interval (AHI) > 6mm
- **Grade 2:** acromio-humeral interval (AHI) 5mm or less
- **Grade 3:** grade 2 with acetabularization of acromion
- **Grade 4:** grade 3 with narrowing of gleno-humeral joint
- **Grade 5:** grade 4 with bony destruction and humeral head collapse

* 144. Please select a treatment

- non-operative (includes medications, injections, physical therapy, or watchful waiting)
- arthroscopic treatment
- hemiarthroplasty
- reverse shoulder arthroplasty

Case 49

An active 65 y.o. who works as a manual laborer presents with moderate signs and symptoms (predominant symptoms that limit most things)

*** 145. Please grade the radiographs according to Seebauer**

- ◯ **Type 1A-Centered, Stable:** minimal superior migration, C-A arch acetabularization and femoralization of humeral head
- ◯ **Type 1B-Centered, Medialized:** minimal superior migration, medial glenoid erosion, C-A arch acetabularization and femoralization of humeral head
- ◯ **Type 2A-Decentered, Limited Stable:** superior migration, superior-medial erosion, extensive C-A arch acetabularization and femoralization of humeral head
- ◯ **Type 2B-Decentered, Unstable:** anterior-superior escape, absent stabilization, C-A arch and anterior structures deficient

*** 146. Please grade the radiographs according to Hamada**

- ◯ **Grade 1:** acromio-humeral interval (AHI) > 6mm
- ◯ **Grade 2:** acromio-humeral interval (AHI) 5mm or less
- ◯ **Grade 3:** grade 2 with acetabularization of acromion
- ◯ **Grade 4:** grade 3 with narrowing of gleno-humeral joint
- ◯ **Grade 5:** grade 4 with bony destruction and humeral head collapse

*** 147. Please select a treatment**

- ◯ non-operative (includes medications, injections, physical therapy, or watchful waiting)
- ◯ arthroscopic treatment
- ◯ hemiarthroplasty
- ◯ reverse shoulder arthroplasty

Case 50

A 65 y.o. who works a desk job and enjoys playing rec basketball and lifting weights presents with moderate signs and symptoms (predominant symptoms that limit most things)

* 148. Please grade the radiographs according to Seebauer

- **Type 1A-Centered, Stable:** minimal superior migration, C-A arch acetabularization and femoralization of humeral head
- **Type 1B-Centered, Medialized:** minimal superior migration, medial glenoid erosion, C-A arch acetabularization and femoralization of humeral head
- **Type 2A-Decentered, Limited Stable:** superior migration, superior-medial erosion, extensive C-A arch acetabularization and femoralization of humeral head
- **Type 2B-Decentered, Unstable:** anterior-superior escape, absent stabilization, C-A arch and anterior structures deficient

* 149. Please grade the radiographs according to Hamada

- **Grade 1:** acromio-humeral interval (AHI) > 6mm
- **Grade 2:** acromio-humeral interval (AHI) 5mm or less
- **Grade 3:** grade 2 with acetabularization of acromion
- **Grade 4:** grade 3 with narrowing of gleno-humeral joint
- **Grade 5:** grade 4 with bony destruction and humeral head collapse

* 150. Please select a treatment

- non-operative (includes medications, injections, physical therapy, or watchful waiting)
- arthroscopic treatment
- hemiarthroplasty
- reverse shoulder arthroplasty

Case 51

An inactive 65 y.o. who is very low demand with their shoulder presents with moderate signs and symptoms (predominant symptoms that limit most things)

* 151. Please grade the radiographs according to Seebauer

- ◯ **Type 1A-Centered, Stable:** minimal superior migration, C-A arch acetabularization and femoralization of humeral head
- ◯ **Type 1B-Centered, Medialized:** minimal superior migration, medial glenoid erosion, C-A arch acetabularization and femoralization of humeral head
- ◯ **Type 2A-Decentered, Limited Stable:** superior migration, superior-medial erosion, extensive C-A arch acetabularization and femoralization of humeral head
- ◯ **Type 2B-Decentered, Unstable:** anterior-superior escape, absent stabilization, C-A arch and anterior structures deficient

* 152. Please grade the radiographs according to Hamada

- ◯ **Grade 1:** acromio-humeral interval (AHI) > 6mm
- ◯ **Grade 2:** acromio-humeral interval (AHI) 5mm or less
- ◯ **Grade 3:** grade 2 with acetabularization of acromion
- ◯ **Grade 4:** grade 3 with narrowing of gleno-humeral joint
- ◯ **Grade 5:** grade 4 with bony destruction and humeral head collapse

* 153. Please select a treatment

- ◯ non-operative (includes medications, injections, physical therapy, or watchful waiting)
- ◯ arthroscopic treatment
- ◯ hemiarthroplasty
- ◯ reverse shoulder arthroplasty

Case 52

An active 65 y.o. who works as a manual laborer presents with severe signs and symptoms (constant)

* 154. Please grade the radiographs according to Seebauer

- ◯ **Type 1A-Centered, Stable:** minimal superior migration, C-A arch acetabularization and femoralization of humeral head
- ◯ **Type 1B-Centered, Medialized:** minimal superior migration, medial glenoid erosion, C-A arch acetabularization and femoralization of humeral head
- ◯ **Type 2A-Decentered, Limited Stable:** superior migration, superior-medial erosion, extensive C-A arch acetabularization and femoralization of humeral head
- ◯ **Type 2B-Decentered, Unstable:** anterior-superior escape, absent stabilization, C-A arch and anterior structures deficient

* 155. Please grade the radiographs according to Hamada

- ◯ **Grade 1:** acromio-humeral interval (AHI) > 6mm
- ◯ **Grade 2:** acromio-humeral interval (AHI) 5mm or less
- ◯ **Grade 3:** grade 2 with acetabularization of acromion
- ◯ **Grade 4:** grade 3 with narrowing of gleno-humeral joint
- ◯ **Grade 5:** grade 4 with bony destruction and humeral head collapse

* 156. Please select a treatment

- ◯ non-operative (includes medications, injections, physical therapy, or watchful waiting)
- ◯ arthroscopic treatment
- ◯ hemiarthroplasty
- ◯ reverse shoulder arthroplasty

Case 53

A 65 y.o. who works a desk job and enjoys playing rec basketball and lifting weights presents with severe signs and symptoms (constant)

*** 157. Please grade the radiographs according to Seebauer**

- ◯ **Type 1A-Centered, Stable:** minimal superior migration, C-A arch acetabularization and femoralization of humeral head
- ◯ **Type 1B-Centered, Medialized:** minimal superior migration, medial glenoid erosion, C-A arch acetabularization and femoralization of humeral head
- ◯ **Type 2A-Decentered, Limited Stable:** superior migration, superior-medial erosion, extensive C-A arch acetabularization and femoralization of humeral head
- ◯ **Type 2B-Decentered, Unstable:** anterior-superior escape, absent stabilization, C-A arch and anterior structures deficient

*** 158. Please grade the radiographs according to Hamada**

- ◯ **Grade 1:** acromio-humeral interval (AHI) > 6mm
- ◯ **Grade 2:** acromio-humeral interval (AHI) 5mm or less
- ◯ **Grade 3:** grade 2 with acetabularization of acromion
- ◯ **Grade 4:** grade 3 with narrowing of gleno-humeral joint
- ◯ **Grade 5:** grade 4 with bony destruction and humeral head collapse

*** 159. Please select a treatment**

- ◯ non-operative (includes medications, injections, physical therapy, or watchful waiting)
- ◯ arthroscopic treatment
- ◯ hemiarthroplasty
- ◯ reverse shoulder arthroplasty

Case 54

An inactive 65 y.o. who is very low demand with their shoulder presents with severe signs and symptoms (constant)

* 160. Please grade the radiographs according to Seebauer

- ◯ **Type 1A-Centered, Stable:** minimal superior migration, C-A arch acetabularization and femoralization of humeral head
- ◯ **Type 1B-Centered, Medialized:** minimal superior migration, medial glenoid erosion, C-A arch acetabularization and femoralization of humeral head
- ◯ **Type 2A-Decentered, Limited Stable:** superior migration, superior-medial erosion, extensive C-A arch acetabularization and femoralization of humeral head
- ◯ **Type 2B-Decentered, Unstable:** anterior-superior escape, absent stabilization, C-A arch and anterior structures deficient

* 161. Please grade the radiographs according to Hamada

- ◯ **Grade 1:** acromio-humeral interval (AHI) > 6mm
- ◯ **Grade 2:** acromio-humeral interval (AHI) 5mm or less
- ◯ **Grade 3:** grade 2 with acetabularization of acromion
- ◯ **Grade 4:** grade 3 with narrowing of gleno-humeral joint
- ◯ **Grade 5:** grade 4 with bony destruction and humeral head collapse

* 162. Please select a treatment

- ◯ non-operative (includes medications, injections, physical therapy, or watchful waiting)
- ◯ arthroscopic treatment
- ◯ hemiarthroplasty
- ◯ reverse shoulder arthroplasty
