# Supplementary material for: Quantifying the Impact of Patient-Specific Factors and Disease Severity on Clinical Decision Making in Cuff Tear Arthropathy: A Case-Based Survey
Source: HSS J. 2019 Jul 5;15(3):276–85. doi: 10.1007/s11420-019-09695-x (PMC6778159; doi:10.1007/s11420-019-09695-x)
Supplement: Supplementary file 2 — (PDF 1409 kb) [file 11420_2019_9695_MOESM2_ESM.pdf]

## Welcome to Our Survey

**This survey contains 54 randomized, short clinical vignettes describing a patient who is presenting with signs and symptoms of cuff tear arthropathy. Assumptions are listed at the top of each page.**

**Each vignette has the same 3 questions:**
**1) Grade the radiographs according to Seebauer**
**2) Grade the radiographs according to Hamada**
**3) Select a treatment**

**Primary Objectives:**
**-Determine the demographics, signs, symptoms, and radiographic features that impact clinical decision making**
**-Determine 1) the reliability of the Seebauer and Hamada grading systems 2) whether or not the grading systems correlate with clinical decision making**

**Thank you for your time!**

## Case 1

**An active 30 y.o. who works as a manual laborer presents with mild signs and symptoms (only during strenuous activities)**

* 1. Please grade the radiographs according to Seebauer

- ◯ **Type 1A-Centered, Stable:** minimal superior migration, C-A arch acetabularization and femoralization of humeral head
- ◯ **Type 1B-Centered, Medialized:** minimal superior migration, medial glenoid erosion, C-A arch acetabularization and femoralization of humeral head
- ◯ **Type 2A-Decentered, Limited Stable:** superior migration, superior-medial erosion, extensive C-A arch acetabularization and femoralization of humeral head
- ◯ **Type 2B-Decentered, Unstable:** anterior-superior escape, absent stabilization, C-A arch and anterior structures deficient

* 2. Please grade the radiographs according to Hamada

- ◯ **Grade 1:** acromio-humeral interval (AHI) > 6mm
- ◯ **Grade 2:** acromio-humeral interval (AHI) 5mm or less
- ◯ **Grade 3:** grade 2 with acetabularization of acromion
- ◯ **Grade 4:** grade 3 with narrowing of gleno-humeral joint
- ◯ **Grade 5:** grade 4 with bony destruction and humeral head collapse

* 3. Please select a treatment

- ◯ non-operative (includes medications, injections, physical therapy, or watchful waiting)
- ◯ arthroscopic treatment
- ◯ hemiarthroplasty
- ◯ reverse shoulder arthroplasty

## Case 2

**A 30 y.o. who works a desk job and enjoys playing rec basketball and lifting weights presents with mild signs and symptoms (only during strenuous activities)**

* 4. Please grade the radiographs according to Seebauer

- **Type 1A-Centered, Stable:** minimal superior migration, C-A arch acetabularization and femoralization of humeral head
- **Type 1B-Centered, Medialized:** minimal superior migration, medial glenoid erosion, C-A arch acetabularization and femoralization of humeral head
- **Type 2A-Decentered, Limited Stable:** superior migration, superior-medial erosion, extensive C-A arch acetabularization and femoralization of humeral head
- **Type 2B-Decentered, Unstable:** anterior-superior escape, absent stabilization, C-A arch and anterior structures deficient

* 5. Please grade the radiographs according to Hamada

- **Grade 1:** acromio-humeral interval (AHI) > 6mm
- **Grade 2:** acromio-humeral interval (AHI) 5mm or less
- **Grade 3:** grade 2 with acetabularization of acromion
- **Grade 4:** grade 3 with narrowing of gleno-humeral joint
- **Grade 5:** grade 4 with bony destruction and humeral head collapse

* 6. Please select a treatment

- non-operative (includes medications, injections, physical therapy, or watchful waiting)
- arthroscopic treatment
- hemiarthroplasty
- reverse shoulder arthroplasty

## Case 3

**An inactive 30 y.o. who is very low demand with their shoulder presents with mild signs and symptoms (only during strenuous activities)**

* 7. Please grade the radiographs according to Seebauer

- ◯ **Type 1A-Centered, Stable:** minimal superior migration, C-A arch acetabularization and femoralization of humeral head
- ◯ **Type 1B-Centered, Medialized:** minimal superior migration, medial glenoid erosion, C-A arch acetabularization and femoralization of humeral head
- ◯ **Type 2A-Decentered, Limited Stable:** superior migration, superior-medial erosion, extensive C-A arch acetabularization and femoralization of humeral head
- ◯ **Type 2B-Decentered, Unstable:** anterior-superior escape, absent stabilization, C-A arch and anterior structures deficient

* 8. Please grade the radiographs according to Hamada

- ◯ **Grade 1:** acromio-humeral interval (AHI) > 6mm
- ◯ **Grade 2:** acromio-humeral interval (AHI) 5mm or less
- ◯ **Grade 3:** grade 2 with acetabularization of acromion
- ◯ **Grade 4:** grade 3 with narrowing of gleno-humeral joint
- ◯ **Grade 5:** grade 4 with bony destruction and humeral head collapse

* 9. Please select a treatment

- ◯ non-operative (includes medications, injections, physical therapy, or watchful waiting)
- ◯ arthroscopic treatment
- ◯ hemiarthroplasty
- ◯ reverse shoulder arthroplasty

## Case 4

**An active 30 y.o. who works as a manual laborer presents with moderate signs and symptoms (predominant symptoms that limit most things)**

* 10. Please grade the radiographs according to Seebauer

- ◯ **Type 1A-Centered, Stable:** minimal superior migration, C-A arch acetabularization and femoralization of humeral head
- ◯ **Type 1B-Centered, Medialized:** minimal superior migration, medial glenoid erosion, C-A arch acetabularization and femoralization of humeral head
- ◯ **Type 2A-Decentered, Limited Stable:** superior migration, superior-medial erosion, extensive C-A arch acetabularization and femoralization of humeral head
- ◯ **Type 2B-Decentered, Unstable:** anterior-superior escape, absent stabilization, C-A arch and anterior structures deficient

* 11. Please grade the radiographs according to Hamada

- ◯ **Grade 1:** acromio-humeral interval (AHI) > 6mm
- ◯ **Grade 2:** acromio-humeral interval (AHI) 5mm or less
- ◯ **Grade 3:** grade 2 with acetabularization of acromion
- ◯ **Grade 4:** grade 3 with narrowing of gleno-humeral joint
- ◯ **Grade 5:** grade 4 with bony destruction and humeral head collapse

* 12. Please select a treatment

- ◯ non-operative (includes medications, injections, physical therapy, or watchful waiting)
- ◯ arthroscopic treatment
- ◯ hemiarthroplasty
- ◯ reverse shoulder arthroplasty

## Case 5

**A 30 y.o. who works a desk job and enjoys playing rec basketball and lifting weights presents with moderate signs and symptoms (predominant symptoms that limit most things)**

* 13. Please grade the radiographs according to Seebauer

- ◯ **Type 1A-Centered, Stable:** minimal superior migration, C-A arch acetabularization and femoralization of humeral head
- ◯ **Type 1B-Centered, Medialized:** minimal superior migration, medial glenoid erosion, C-A arch acetabularization and femoralization of humeral head
- ◯ **Type 2A-Decentered, Limited Stable:** superior migration, superior-medial erosion, extensive C-A arch acetabularization and femoralization of humeral head
- ◯ **Type 2B-Decentered, Unstable:** anterior-superior escape, absent stabilization, C-A arch and anterior structures deficient

* 14. Please grade the radiographs according to Hamada

- ◯ **Grade 1:** acromio-humeral interval (AHI) > 6mm
- ◯ **Grade 2:** acromio-humeral interval (AHI) 5mm or less
- ◯ **Grade 3:** grade 2 with acetabularization of acromion
- ◯ **Grade 4:** grade 3 with narrowing of gleno-humeral joint
- ◯ **Grade 5:** grade 4 with bony destruction and humeral head collapse

* 15. Please select a treatment

- ◯ non-operative (includes medications, injections, physical therapy, or watchful waiting)
- ◯ arthroscopic treatment
- ◯ hemiarthroplasty
- ◯ reverse shoulder arthroplasty

## Case 6

**An inactive 30 y.o. who is very low demand with their shoulder presents with moderate signs and symptoms (predominant symptoms that limit most things)**

* 16. Please grade the radiographs according to Seebauer

- ◯ **Type 1A-Centered, Stable:** minimal superior migration, C-A arch acetabularization and femoralization of humeral head
- ◯ **Type 1B-Centered, Medialized:** minimal superior migration, medial glenoid erosion, C-A arch acetabularization and femoralization of humeral head
- ◯ **Type 2A-Decentered, Limited Stable:** superior migration, superior-medial erosion, extensive C-A arch acetabularization and femoralization of humeral head
- ◯ **Type 2B-Decentered, Unstable:** anterior-superior escape, absent stabilization, C-A arch and anterior structures deficient

* 17. Please grade the radiographs according to Hamada

- ◯ **Grade 1:** acromio-humeral interval (AHI) > 6mm
- ◯ **Grade 2:** acromio-humeral interval (AHI) 5mm or less
- ◯ **Grade 3:** grade 2 with acetabularization of acromion
- ◯ **Grade 4:** grade 3 with narrowing of gleno-humeral joint
- ◯ **Grade 5:** grade 4 with bony destruction and humeral head collapse

* 18. Please select a treatment

- ◯ non-operative (includes medications, injections, physical therapy, or watchful waiting)
- ◯ arthroscopic treatment
- ◯ hemiarthroplasty
- ◯ reverse shoulder arthroplasty

## Case 7

**An active 30 y.o. who works as a manual laborer presents with severe signs and symptoms (constant)**

* 19. Please grade the radiographs according to Seebauer

- **Type 1A-Centered, Stable:** minimal superior migration, C-A arch acetabularization and femoralization of humeral head
- **Type 1B-Centered, Medialized:** minimal superior migration, medial glenoid erosion, C-A arch acetabularization and femoralization of humeral head
- **Type 2A-Decentered, Limited Stable:** superior migration, superior-medial erosion, extensive C-A arch acetabularization and femoralization of humeral head
- **Type 2B-Decentered, Unstable:** anterior-superior escape, absent stabilization, C-A arch and anterior structures deficient

* 20. Please grade the radiographs according to Hamada

- **Grade 1:** acromio-humeral interval (AHI) > 6mm
- **Grade 2:** acromio-humeral interval (AHI) 5mm or less
- **Grade 3:** grade 2 with acetabularization of acromion
- **Grade 4:** grade 3 with narrowing of gleno-humeral joint
- **Grade 5:** grade 4 with bony destruction and humeral head collapse

* 21. Please select a treatment

- non-operative (includes medications, injections, physical therapy, or watchful waiting)
- arthroscopic treatment
- hemiarthroplasty
- reverse shoulder arthroplasty

## Case 8

**A 30 y.o. who works a desk job and enjoys playing rec basketball and lifting weights presents with severe signs and symptoms (constant)**

* 22. Please grade the radiographs according to Seebauer

- **Type 1A-Centered, Stable:** minimal superior migration, C-A arch acetabularization and femoralization of humeral head
- **Type 1B-Centered, Medialized:** minimal superior migration, medial glenoid erosion, C-A arch acetabularization and femoralization of humeral head
- **Type 2A-Decentered, Limited Stable:** superior migration, superior-medial erosion, extensive C-A arch acetabularization and femoralization of humeral head
- **Type 2B-Decentered, Unstable:** anterior-superior escape, absent stabilization, C-A arch and anterior structures deficient

* 23. Please grade the radiographs according to Hamada

- **Grade 1:** acromio-humeral interval (AHI) > 6mm
- **Grade 2:** acromio-humeral interval (AHI) 5mm or less
- **Grade 3:** grade 2 with acetabularization of acromion
- **Grade 4:** grade 3 with narrowing of gleno-humeral joint
- **Grade 5:** grade 4 with bony destruction and humeral head collapse

* 24. Please select a treatment

- non-operative (includes medications, injections, physical therapy, or watchful waiting)
- arthroscopic treatment
- hemiarthroplasty
- reverse shoulder arthroplasty

## Case 9

**An inactive 30 y.o. who is very low demand with their shoulder presents with severe signs and symptoms (constant)**

* 25. Please grade the radiographs according to Seebauer

- ◯ **Type 1A-Centered, Stable:** minimal superior migration, C-A arch acetabularization and femoralization of humeral head
- ◯ **Type 1B-Centered, Medialized:** minimal superior migration, medial glenoid erosion, C-A arch acetabularization and femoralization of humeral head
- ◯ **Type 2A-Decentered, Limited Stable:** superior migration, superior-medial erosion, extensive C-A arch acetabularization and femoralization of humeral head
- ◯ **Type 2B-Decentered, Unstable:** anterior-superior escape, absent stabilization, C-A arch and anterior structures deficient

* 26. Please grade the radiographs according to Hamada

- ◯ **Grade 1:** acromio-humeral interval (AHI) > 6mm
- ◯ **Grade 2:** acromio-humeral interval (AHI) 5mm or less
- ◯ **Grade 3:** grade 2 with acetabularization of acromion
- ◯ **Grade 4:** grade 3 with narrowing of gleno-humeral joint
- ◯ **Grade 5:** grade 4 with bony destruction and humeral head collapse

* 27. Please select a treatment

- ◯ non-operative (includes medications, injections, physical therapy, or watchful waiting)
- ◯ arthroscopic treatment
- ◯ hemiarthroplasty
- ◯ reverse shoulder arthroplasty

## Case 10

**An active 45 y.o. who works as a manual laborer presents with mild signs and symptoms (only during strenuous activities)**

* 28. Please grade the radiographs according to Seebauer

- ◯ **Type 1A-Centered, Stable:** minimal superior migration, C-A arch acetabularization and femoralization of humeral head
- ◯ **Type 1B-Centered, Medialized:** minimal superior migration, medial glenoid erosion, C-A arch acetabularization and femoralization of humeral head
- ◯ **Type 2A-Decentered, Limited Stable:** superior migration, superior-medial erosion, extensive C-A arch acetabularization and femoralization of humeral head
- ◯ **Type 2B-Decentered, Unstable:** anterior-superior escape, absent stabilization, C-A arch and anterior structures deficient

* 29. Please grade the radiographs according to Hamada

- ◯ **Grade 1:** acromio-humeral interval (AHI) > 6mm
- ◯ **Grade 2:** acromio-humeral interval (AHI) 5mm or less
- ◯ **Grade 3:** grade 2 with acetabularization of acromion
- ◯ **Grade 4:** grade 3 with narrowing of gleno-humeral joint
- ◯ **Grade 5:** grade 4 with bony destruction and humeral head collapse

* 30. Please select a treatment

- ◯ non-operative (includes medications, injections, physical therapy, or watchful waiting)
- ◯ arthroscopic treatment
- ◯ hemiarthroplasty
- ◯ reverse shoulder arthroplasty

## Case 11

**A 45 y.o. who works a desk job and enjoys playing rec basketball and lifting weights presents with mild signs and symptoms (only during strenuous activities)**

* 31. Please grade the radiographs according to Seebauer

- ◯ **Type 1A-Centered, Stable:** minimal superior migration, C-A arch acetabularization and femoralization of humeral head
- ◯ **Type 1B-Centered, Medialized:** minimal superior migration, medial glenoid erosion, C-A arch acetabularization and femoralization of humeral head
- ◯ **Type 2A-Decentered, Limited Stable:** superior migration, superior-medial erosion, extensive C-A arch acetabularization and femoralization of humeral head
- ◯ **Type 2B-Decentered, Unstable:** anterior-superior escape, absent stabilization, C-A arch and anterior structures deficient

* 32. Please grade the radiographs according to Hamada

- ◯ **Grade 1:** acromio-humeral interval (AHI) > 6mm
- ◯ **Grade 2:** acromio-humeral interval (AHI) 5mm or less
- ◯ **Grade 3:** grade 2 with acetabularization of acromion
- ◯ **Grade 4:** grade 3 with narrowing of gleno-humeral joint
- ◯ **Grade 5:** grade 4 with bony destruction and humeral head collapse

* 33. Please select a treatment

- ◯ non-operative (includes medications, injections, physical therapy, or watchful waiting)
- ◯ arthroscopic treatment
- ◯ hemiarthroplasty
- ◯ reverse shoulder arthroplasty

## Case 12

**An inactive 45 y.o. who is very low demand with their shoulder presents with mild signs and symptoms (only during strenuous activities)**

* 34. Please grade the radiographs according to Seebauer

- ◯ **Type 1A-Centered, Stable:** minimal superior migration, C-A arch acetabularization and femoralization of humeral head
- ◯ **Type 1B-Centered, Medialized:** minimal superior migration, medial glenoid erosion, C-A arch acetabularization and femoralization of humeral head
- ◯ **Type 2A-Decentered, Limited Stable:** superior migration, superior-medial erosion, extensive C-A arch acetabularization and femoralization of humeral head
- ◯ **Type 2B-Decentered, Unstable:** anterior-superior escape, absent stabilization, C-A arch and anterior structures deficient

* 35. Please grade the radiographs according to Hamada

- ◯ **Grade 1:** acromio-humeral interval (AHI) > 6mm
- ◯ **Grade 2:** acromio-humeral interval (AHI) 5mm or less
- ◯ **Grade 3:** grade 2 with acetabularization of acromion
- ◯ **Grade 4:** grade 3 with narrowing of gleno-humeral joint
- ◯ **Grade 5:** grade 4 with bony destruction and humeral head collapse

* 36. Please select a treatment

- ◯ non-operative (includes medications, injections, physical therapy, or watchful waiting)
- ◯ arthroscopic treatment
- ◯ hemiarthroplasty
- ◯ reverse shoulder arthroplasty

## Case 13

**An active 45 y.o. who works as a manual laborer presents with moderate signs and symptoms (predominant symptoms that limit most things)**

* 37. Please grade the radiographs according to Seebauer

- ◯ **Type 1A-Centered, Stable:** minimal superior migration, C-A arch acetabularization and femoralization of humeral head
- ◯ **Type 1B-Centered, Medialized:** minimal superior migration, medial glenoid erosion, C-A arch acetabularization and femoralization of humeral head
- ◯ **Type 2A-Decentered, Limited Stable:** superior migration, superior-medial erosion, extensive C-A arch acetabularization and femoralization of humeral head
- ◯ **Type 2B-Decentered, Unstable:** anterior-superior escape, absent stabilization, C-A arch and anterior structures deficient

* 38. Please grade the radiographs according to Hamada

- ◯ **Grade 1:** acromio-humeral interval (AHI) > 6mm
- ◯ **Grade 2:** acromio-humeral interval (AHI) 5mm or less
- ◯ **Grade 3:** grade 2 with acetabularization of acromion
- ◯ **Grade 4:** grade 3 with narrowing of gleno-humeral joint
- ◯ **Grade 5:** grade 4 with bony destruction and humeral head collapse

* 39. Please select a treatment

- ◯ non-operative (includes medications, injections, physical therapy, or watchful waiting)
- ◯ arthroscopic treatment
- ◯ hemiarthroplasty
- ◯ reverse shoulder arthroplasty

## Case 14

**A 45 y.o. who works a desk job and enjoys playing rec basketball and lifting weights presents with moderate signs and symptoms (predominant symptoms that limit most things)**

* 40. Please grade the radiographs according to Seebauer

- ◯ **Type 1A-Centered, Stable:** minimal superior migration, C-A arch acetabularization and femoralization of humeral head
- ◯ **Type 1B-Centered, Medialized:** minimal superior migration, medial glenoid erosion, C-A arch acetabularization and femoralization of humeral head
- ◯ **Type 2A-Decentered, Limited Stable:** superior migration, superior-medial erosion, extensive C-A arch acetabularization and femoralization of humeral head
- ◯ **Type 2B-Decentered, Unstable:** anterior-superior escape, absent stabilization, C-A arch and anterior structures deficient

* 41. Please grade the radiographs according to Hamada

- ◯ **Grade 1:** acromio-humeral interval (AHI) > 6mm
- ◯ **Grade 2:** acromio-humeral interval (AHI) 5mm or less
- ◯ **Grade 3:** grade 2 with acetabularization of acromion
- ◯ **Grade 4:** grade 3 with narrowing of gleno-humeral joint
- ◯ **Grade 5:** grade 4 with bony destruction and humeral head collapse

* 42. Please select a treatment

- ◯ non-operative (includes medications, injections, physical therapy, or watchful waiting)
- ◯ arthroscopic treatment
- ◯ hemiarthroplasty
- ◯ reverse shoulder arthroplasty

## Case 15

**An inactive 45 y.o. who is very low demand with their shoulder presents with moderate signs and symptoms (predominant symptoms that limit most things)**

* 43. Please grade the radiographs according to Seebauer

- ◯ **Type 1A-Centered, Stable:** minimal superior migration, C-A arch acetabularization and femoralization of humeral head
- ◯ **Type 1B-Centered, Medialized:** minimal superior migration, medial glenoid erosion, C-A arch acetabularization and femoralization of humeral head
- ◯ **Type 2A-Decentered, Limited Stable:** superior migration, superior-medial erosion, extensive C-A arch acetabularization and femoralization of humeral head
- ◯ **Type 2B-Decentered, Unstable:** anterior-superior escape, absent stabilization, C-A arch and anterior structures deficient

* 44. Please grade the radiographs according to Hamada

- ◯ **Grade 1:** acromio-humeral interval (AHI) > 6mm
- ◯ **Grade 2:** acromio-humeral interval (AHI) 5mm or less
- ◯ **Grade 3:** grade 2 with acetabularization of acromion
- ◯ **Grade 4:** grade 3 with narrowing of gleno-humeral joint
- ◯ **Grade 5:** grade 4 with bony destruction and humeral head collapse

* 45. Please select a treatment

- ◯ non-operative (includes medications, injections, physical therapy, or watchful waiting)
- ◯ arthroscopic treatment
- ◯ hemiarthroplasty
- ◯ reverse shoulder arthroplasty

## Case 16

**An active 45 y.o. who works as a manual laborer presents with severe signs and symptoms (constant)**

* 46. Please grade the radiographs according to Seebauer

- **Type 1A-Centered, Stable:** minimal superior migration, C-A arch acetabularization and femoralization of humeral head
- **Type 1B-Centered, Medialized:** minimal superior migration, medial glenoid erosion, C-A arch acetabularization and femoralization of humeral head
- **Type 2A-Decentered, Limited Stable:** superior migration, superior-medial erosion, extensive C-A arch acetabularization and femoralization of humeral head
- **Type 2B-Decentered, Unstable:** anterior-superior escape, absent stabilization, C-A arch and anterior structures deficient

* 47. Please grade the radiographs according to Hamada

- **Grade 1:** acromio-humeral interval (AHI) > 6mm
- **Grade 2:** acromio-humeral interval (AHI) 5mm or less
- **Grade 3:** grade 2 with acetabularization of acromion
- **Grade 4:** grade 3 with narrowing of gleno-humeral joint
- **Grade 5:** grade 4 with bony destruction and humeral head collapse

* 48. Please select a treatment

- non-operative (includes medications, injections, physical therapy, or watchful waiting)
- arthroscopic treatment
- hemiarthroplasty
- reverse shoulder arthroplasty

## Case 17

**A 45 y.o. who works a desk job and enjoys playing rec basketball and lifting weights presents with severe signs and symptoms (constant)**

* 49. Please grade the radiographs according to Seebauer

- ◯ **Type 1A-Centered, Stable:** minimal superior migration, C-A arch acetabularization and femoralization of humeral head
- ◯ **Type 1B-Centered, Medialized:** minimal superior migration, medial glenoid erosion, C-A arch acetabularization and femoralization of humeral head
- ◯ **Type 2A-Decentered, Limited Stable:** superior migration, superior-medial erosion, extensive C-A arch acetabularization and femoralization of humeral head
- ◯ **Type 2B-Decentered, Unstable:** anterior-superior escape, absent stabilization, C-A arch and anterior structures deficient

* 50. Please grade the radiographs according to Hamada

- ◯ **Grade 1:** acromio-humeral interval (AHI) > 6mm
- ◯ **Grade 2:** acromio-humeral interval (AHI) 5mm or less
- ◯ **Grade 3:** grade 2 with acetabularization of acromion
- ◯ **Grade 4:** grade 3 with narrowing of gleno-humeral joint
- ◯ **Grade 5:** grade 4 with bony destruction and humeral head collapse

* 51. Please select a treatment

- ◯ non-operative (includes medications, injections, physical therapy, or watchful waiting)
- ◯ arthroscopic treatment
- ◯ hemiarthroplasty
- ◯ reverse shoulder arthroplasty

## Case 18

**An inactive 45 y.o. who is very low demand with their shoulder presents with severe signs and symptoms (constant)**

* 52. Please grade the radiographs according to Seebauer

- **Type 1A-Centered, Stable:** minimal superior migration, C-A arch acetabularization and femoralization of humeral head
- **Type 1B-Centered, Medialized:** minimal superior migration, medial glenoid erosion, C-A arch acetabularization and femoralization of humeral head
- **Type 2A-Decentered, Limited Stable:** superior migration, superior-medial erosion, extensive C-A arch acetabularization and femoralization of humeral head
- **Type 2B-Decentered, Unstable:** anterior-superior escape, absent stabilization, C-A arch and anterior structures deficient

* 53. Please grade the radiographs according to Hamada

- **Grade 1:** acromio-humeral interval (AHI) > 6mm
- **Grade 2:** acromio-humeral interval (AHI) 5mm or less
- **Grade 3:** grade 2 with acetabularization of acromion
- **Grade 4:** grade 3 with narrowing of gleno-humeral joint
- **Grade 5:** grade 4 with bony destruction and humeral head collapse

* 54. Please select a treatment

- non-operative (includes medications, injections, physical therapy, or watchful waiting)
- arthroscopic treatment
- hemiarthroplasty
- reverse shoulder arthroplasty

## Case 19

**An active 65 y.o. who works as a manual laborer presents with mild signs and symptoms (only during strenuous activities)**

* 55. Please grade the radiographs according to Seebauer

- ◯ **Type 1A-Centered, Stable:** minimal superior migration, C-A arch acetabularization and femoralization of humeral head
- ◯ **Type 1B-Centered, Medialized:** minimal superior migration, medial glenoid erosion, C-A arch acetabularization and femoralization of humeral head
- ◯ **Type 2A-Decentered, Limited Stable:** superior migration, superior-medial erosion, extensive C-A arch acetabularization and femoralization of humeral head
- ◯ **Type 2B-Decentered, Unstable:** anterior-superior escape, absent stabilization, C-A arch and anterior structures deficient

* 56. Please grade the radiographs according to Hamada

- ◯ **Grade 1:** acromio-humeral interval (AHI) > 6mm
- ◯ **Grade 2:** acromio-humeral interval (AHI) 5mm or less
- ◯ **Grade 3:** grade 2 with acetabularization of acromion
- ◯ **Grade 4:** grade 3 with narrowing of gleno-humeral joint
- ◯ **Grade 5:** grade 4 with bony destruction and humeral head collapse

* 57. Please select a treatment

- ◯ non-operative (includes medications, injections, physical therapy, or watchful waiting)
- ◯ arthroscopic treatment
- ◯ hemiarthroplasty
- ◯ reverse shoulder arthroplasty

## Case 20

**A 65 y.o. who works a desk job and enjoys playing rec basketball and lifting weights presents with mild signs and symptoms (only during strenuous activities)**

* 58. Please grade the radiographs according to Seebauer

- **Type 1A-Centered, Stable:** minimal superior migration, C-A arch acetabularization and femoralization of humeral head
- **Type 1B-Centered, Medialized:** minimal superior migration, medial glenoid erosion, C-A arch acetabularization and femoralization of humeral head
- **Type 2A-Decentered, Limited Stable:** superior migration, superior-medial erosion, extensive C-A arch acetabularization and femoralization of humeral head
- **Type 2B-Decentered, Unstable:** anterior-superior escape, absent stabilization, C-A arch and anterior structures deficient

* 59. Please grade the radiographs according to Hamada

- **Grade 1:** acromio-humeral interval (AHI) > 6mm
- **Grade 2:** acromio-humeral interval (AHI) 5mm or less
- **Grade 3:** grade 2 with acetabularization of acromion
- **Grade 4:** grade 3 with narrowing of gleno-humeral joint
- **Grade 5:** grade 4 with bony destruction and humeral head collapse

* 60. Please select a treatment

- non-operative (includes medications, injections, physical therapy, or watchful waiting)
- arthroscopic treatment
- hemiarthroplasty
- reverse shoulder arthroplasty

## Case 21

**An inactive 65 y.o. who is very low demand with their shoulder presents with mild signs and symptoms (only during strenuous activities)**

* 61. Please grade the radiographs according to Seebauer

- ◯ **Type 1A-Centered, Stable:** minimal superior migration, C-A arch acetabularization and femoralization of humeral head
- ◯ **Type 1B-Centered, Medialized:** minimal superior migration, medial glenoid erosion, C-A arch acetabularization and femoralization of humeral head
- ◯ **Type 2A-Decentered, Limited Stable:** superior migration, superior-medial erosion, extensive C-A arch acetabularization and femoralization of humeral head
- ◯ **Type 2B-Decentered, Unstable:** anterior-superior escape, absent stabilization, C-A arch and anterior structures deficient

* 62. Please grade the radiographs according to Hamada

- ◯ **Grade 1:** acromio-humeral interval (AHI) > 6mm
- ◯ **Grade 2:** acromio-humeral interval (AHI) 5mm or less
- ◯ **Grade 3:** grade 2 with acetabularization of acromion
- ◯ **Grade 4:** grade 3 with narrowing of gleno-humeral joint
- ◯ **Grade 5:** grade 4 with bony destruction and humeral head collapse

* 63. Please select a treatment

- ◯ non-operative (includes medications, injections, physical therapy, or watchful waiting)
- ◯ arthroscopic treatment
- ◯ hemiarthroplasty
- ◯ reverse shoulder arthroplasty

## Case 22

**An active 65 y.o. who works as a manual laborer presents with moderate signs and symptoms (predominant symptoms that limit most things)**

* 64. Please grade the radiographs according to Seebauer

- ◯ **Type 1A-Centered, Stable:** minimal superior migration, C-A arch acetabularization and femoralization of humeral head
- ◯ **Type 1B-Centered, Medialized:** minimal superior migration, medial glenoid erosion, C-A arch acetabularization and femoralization of humeral head
- ◯ **Type 2A-Decentered, Limited Stable:** superior migration, superior-medial erosion, extensive C-A arch acetabularization and femoralization of humeral head
- ◯ **Type 2B-Decentered, Unstable:** anterior-superior escape, absent stabilization, C-A arch and anterior structures deficient

* 65. Please grade the radiographs according to Hamada

- ◯ **Grade 1:** acromio-humeral interval (AHI) > 6mm
- ◯ **Grade 2:** acromio-humeral interval (AHI) 5mm or less
- ◯ **Grade 3:** grade 2 with acetabularization of acromion
- ◯ **Grade 4:** grade 3 with narrowing of gleno-humeral joint
- ◯ **Grade 5:** grade 4 with bony destruction and humeral head collapse

* 66. Please select a treatment

- ◯ non-operative (includes medications, injections, physical therapy, or watchful waiting)
- ◯ arthroscopic treatment
- ◯ hemiarthroplasty
- ◯ reverse shoulder arthroplasty

## Case 23

**A 65 y.o. who works a desk job and enjoys playing rec basketball and lifting weights presents with moderate signs and symptoms (predominant symptoms that limit most things)**

* 67. Please grade the radiographs according to Seebauer

- ◯ **Type 1A-Centered, Stable:** minimal superior migration, C-A arch acetabularization and femoralization of humeral head
- ◯ **Type 1B-Centered, Medialized:** minimal superior migration, medial glenoid erosion, C-A arch acetabularization and femoralization of humeral head
- ◯ **Type 2A-Decentered, Limited Stable:** superior migration, superior-medial erosion, extensive C-A arch acetabularization and femoralization of humeral head
- ◯ **Type 2B-Decentered, Unstable:** anterior-superior escape, absent stabilization, C-A arch and anterior structures deficient

* 68. Please grade the radiographs according to Hamada

- ◯ **Grade 1:** acromio-humeral interval (AHI) > 6mm
- ◯ **Grade 2:** acromio-humeral interval (AHI) 5mm or less
- ◯ **Grade 3:** grade 2 with acetabularization of acromion
- ◯ **Grade 4:** grade 3 with narrowing of gleno-humeral joint
- ◯ **Grade 5:** grade 4 with bony destruction and humeral head collapse

* 69. Please select a treatment

- ◯ non-operative (includes medications, injections, physical therapy, or watchful waiting)
- ◯ arthroscopic treatment
- ◯ hemiarthroplasty
- ◯ reverse shoulder arthroplasty

## Case 24

**An inactive 65 y.o. who is very low demand with their shoulder presents with moderate signs and symptoms (predominant symptoms that limit most things)**

* 70. Please grade the radiographs according to Seebauer

- ◯ **Type 1A-Centered, Stable:** minimal superior migration, C-A arch acetabularization and femoralization of humeral head
- ◯ **Type 1B-Centered, Medialized:** minimal superior migration, medial glenoid erosion, C-A arch acetabularization and femoralization of humeral head
- ◯ **Type 2A-Decentered, Limited Stable:** superior migration, superior-medial erosion, extensive C-A arch acetabularization and femoralization of humeral head
- ◯ **Type 2B-Decentered, Unstable:** anterior-superior escape, absent stabilization, C-A arch and anterior structures deficient

* 71. Please grade the radiographs according to Hamada

- ◯ **Grade 1:** acromio-humeral interval (AHI) > 6mm
- ◯ **Grade 2:** acromio-humeral interval (AHI) 5mm or less
- ◯ **Grade 3:** grade 2 with acetabularization of acromion
- ◯ **Grade 4:** grade 3 with narrowing of gleno-humeral joint
- ◯ **Grade 5:** grade 4 with bony destruction and humeral head collapse

* 72. Please select a treatment

- ◯ non-operative (includes medications, injections, physical therapy, or watchful waiting)
- ◯ arthroscopic treatment
- ◯ hemiarthroplasty
- ◯ reverse shoulder arthroplasty

## Case 25

**An active 65 y.o. who works as a manual laborer presents with severe signs and symptoms (constant)**

* 73. Please grade the radiographs according to Seebauer

- ◯ **Type 1A-Centered, Stable:** minimal superior migration, C-A arch acetabularization and femoralization of humeral head
- ◯ **Type 1B-Centered, Medialized:** minimal superior migration, medial glenoid erosion, C-A arch acetabularization and femoralization of humeral head
- ◯ **Type 2A-Decentered, Limited Stable:** superior migration, superior-medial erosion, extensive C-A arch acetabularization and femoralization of humeral head
- ◯ **Type 2B-Decentered, Unstable:** anterior-superior escape, absent stabilization, C-A arch and anterior structures deficient

* 74. Please grade the radiographs according to Hamada

- ◯ **Grade 1:** acromio-humeral interval (AHI) > 6mm
- ◯ **Grade 2:** acromio-humeral interval (AHI) 5mm or less
- ◯ **Grade 3:** grade 2 with acetabularization of acromion
- ◯ **Grade 4:** grade 3 with narrowing of gleno-humeral joint
- ◯ **Grade 5:** grade 4 with bony destruction and humeral head collapse

* 75. Please select a treatment

- ◯ non-operative (includes medications, injections, physical therapy, or watchful waiting)
- ◯ arthroscopic treatment
- ◯ hemiarthroplasty
- ◯ reverse shoulder arthroplasty

## Case 26

**A 65 y.o. who works a desk job and enjoys playing rec basketball and lifting weights presents with severe signs and symptoms (constant)**

* 76. Please grade the radiographs according to Seebauer

- ○ **Type 1A-Centered, Stable:** minimal superior migration, C-A arch acetabularization and femoralization of humeral head
- ○ **Type 1B-Centered, Medialized:** minimal superior migration, medial glenoid erosion, C-A arch acetabularization and femoralization of humeral head
- ○ **Type 2A-Decentered, Limited Stable:** superior migration, superior-medial erosion, extensive C-A arch acetabularization and femoralization of humeral head
- ○ **Type 2B-Decentered, Unstable:** anterior-superior escape, absent stabilization, C-A arch and anterior structures deficient

* 77. Please grade the radiographs according to Hamada

- ○ **Grade 1:** acromio-humeral interval (AHI) > 6mm
- ○ **Grade 2:** acromio-humeral interval (AHI) 5mm or less
- ○ **Grade 3:** grade 2 with acetabularization of acromion
- ○ **Grade 4:** grade 3 with narrowing of gleno-humeral joint
- ○ **Grade 5:** grade 4 with bony destruction and humeral head collapse

* 78. Please select a treatment

- ○ non-operative (includes medications, injections, physical therapy, or watchful waiting)
- ○ arthroscopic treatment
- ○ hemiarthroplasty
- ○ reverse shoulder arthroplasty

## Case 27

**An inactive 65 y.o. who is very low demand with their shoulder presents with severe signs and symptoms (constant)**

* 79. Please grade the radiographs according to Seebauer

- **Type 1A-Centered, Stable:** minimal superior migration, C-A arch acetabularization and femoralization of humeral head
- **Type 1B-Centered, Medialized:** minimal superior migration, medial glenoid erosion, C-A arch acetabularization and femoralization of humeral head
- **Type 2A-Decentered, Limited Stable:** superior migration, superior-medial erosion, extensive C-A arch acetabularization and femoralization of humeral head
- **Type 2B-Decentered, Unstable:** anterior-superior escape, absent stabilization, C-A arch and anterior structures deficient

* 80. Please grade the radiographs according to Hamada

- **Grade 1:** acromio-humeral interval (AHI) > 6mm
- **Grade 2:** acromio-humeral interval (AHI) 5mm or less
- **Grade 3:** grade 2 with acetabularization of acromion
- **Grade 4:** grade 3 with narrowing of gleno-humeral joint
- **Grade 5:** grade 4 with bony destruction and humeral head collapse

* 81. Please select a treatment

- non-operative (includes medications, injections, physical therapy, or watchful waiting)
- arthroscopic treatment
- hemiarthroplasty
- reverse shoulder arthroplasty

## Case 28

**An active 30 y.o. who works as a manual laborer presents with mild signs and symptoms (only during strenuous activities)**

* 82. Please grade the radiographs according to Seebauer

- ◯ **Type 1A-Centered, Stable:** minimal superior migration, C-A arch acetabularization and femoralization of humeral head
- ◯ **Type 1B-Centered, Medialized:** minimal superior migration, medial glenoid erosion, C-A arch acetabularization and femoralization of humeral head
- ◯ **Type 2A-Decentered, Limited Stable:** superior migration, superior-medial erosion, extensive C-A arch acetabularization and femoralization of humeral head
- ◯ **Type 2B-Decentered, Unstable:** anterior-superior escape, absent stabilization, C-A arch and anterior structures deficient

* 83. Please grade the radiographs according to Hamada

- ◯ **Grade 1:** acromio-humeral interval (AHI) > 6mm
- ◯ **Grade 2:** acromio-humeral interval (AHI) 5mm or less
- ◯ **Grade 3:** grade 2 with acetabularization of acromion
- ◯ **Grade 4:** grade 3 with narrowing of gleno-humeral joint
- ◯ **Grade 5:** grade 4 with bony destruction and humeral head collapse

* 84. Please select a treatment

- ◯ non-operative (includes medications, injections, physical therapy, or watchful waiting)
- ◯ arthroscopic treatment
- ◯ hemiarthroplasty
- ◯ reverse shoulder arthroplasty

## Case 29

**A 30 y.o. who works a desk job and enjoys playing rec basketball and lifting weights presents with mild signs and symptoms (only during strenuous activities)**

* 85. Please grade the radiographs according to Seebauer

- ◯ **Type 1A-Centered, Stable:** minimal superior migration, C-A arch acetabularization and femoralization of humeral head
- ◯ **Type 1B-Centered, Medialized:** minimal superior migration, medial glenoid erosion, C-A arch acetabularization and femoralization of humeral head
- ◯ **Type 2A-Decentered, Limited Stable:** superior migration, superior-medial erosion, extensive C-A arch acetabularization and femoralization of humeral head
- ◯ **Type 2B-Decentered, Unstable:** anterior-superior escape, absent stabilization, C-A arch and anterior structures deficient

* 86. Please grade the radiographs according to Hamada

- ◯ **Grade 1:** acromio-humeral interval (AHI) > 6mm
- ◯ **Grade 2:** acromio-humeral interval (AHI) 5mm or less
- ◯ **Grade 3:** grade 2 with acetabularization of acromion
- ◯ **Grade 4:** grade 3 with narrowing of gleno-humeral joint
- ◯ **Grade 5:** grade 4 with bony destruction and humeral head collapse

* 87. Please select a treatment

- ◯ non-operative (includes medications, injections, physical therapy, or watchful waiting)
- ◯ arthroscopic treatment
- ◯ hemiarthroplasty
- ◯ reverse shoulder arthroplasty

## Case 30

**An inactive 30 y.o. who is very low demand with their shoulder presents with mild signs and symptoms (only during strenuous activities)**

* 88. Please grade the radiographs according to Seebauer

- **Type 1A-Centered, Stable:** minimal superior migration, C-A arch acetabularization and femoralization of humeral head
- **Type 1B-Centered, Medialized:** minimal superior migration, medial glenoid erosion, C-A arch acetabularization and femoralization of humeral head
- **Type 2A-Decentered, Limited Stable:** superior migration, superior-medial erosion, extensive C-A arch acetabularization and femoralization of humeral head
- **Type 2B-Decentered, Unstable:** anterior-superior escape, absent stabilization, C-A arch and anterior structures deficient

* 89. Please grade the radiographs according to Hamada

- **Grade 1:** acromio-humeral interval (AHI) > 6mm
- **Grade 2:** acromio-humeral interval (AHI) 5mm or less
- **Grade 3:** grade 2 with acetabularization of acromion
- **Grade 4:** grade 3 with narrowing of gleno-humeral joint
- **Grade 5:** grade 4 with bony destruction and humeral head collapse

* 90. Please select a treatment

- non-operative (includes medications, injections, physical therapy, or watchful waiting)
- arthroscopic treatment
- hemiarthroplasty
- reverse shoulder arthroplasty

## Case 31

**An active 30 y.o. who works as a manual laborer presents with moderate signs and symptoms (predominant symptoms that limit most things)**

* 91. Please grade the radiographs according to Seebauer

- **Type 1A-Centered, Stable:** minimal superior migration, C-A arch acetabularization and femoralization of humeral head
- **Type 1B-Centered, Medialized:** minimal superior migration, medial glenoid erosion, C-A arch acetabularization and femoralization of humeral head
- **Type 2A-Decentered, Limited Stable:** superior migration, superior-medial erosion, extensive C-A arch acetabularization and femoralization of humeral head
- **Type 2B-Decentered, Unstable:** anterior-superior escape, absent stabilization, C-A arch and anterior structures deficient

* 92. Please grade the radiographs according to Hamada

- **Grade 1:** acromio-humeral interval (AHI) > 6mm
- **Grade 2:** acromio-humeral interval (AHI) 5mm or less
- **Grade 3:** grade 2 with acetabularization of acromion
- **Grade 4:** grade 3 with narrowing of gleno-humeral joint
- **Grade 5:** grade 4 with bony destruction and humeral head collapse

* 93. Please select a treatment

- non-operative (includes medications, injections, physical therapy, or watchful waiting)
- arthroscopic treatment
- hemiarthroplasty
- reverse shoulder arthroplasty

## Case 32

**A 30 y.o. who works a desk job and enjoys playing rec basketball and lifting weights presents with moderate signs and symptoms (predominant symptoms that limit most things)**

* 94. Please grade the radiographs according to Seebauer

- ◯ **Type 1A-Centered, Stable:** minimal superior migration, C-A arch acetabularization and femoralization of humeral head
- ◯ **Type 1B-Centered, Medialized:** minimal superior migration, medial glenoid erosion, C-A arch acetabularization and femoralization of humeral head
- ◯ **Type 2A-Decentered, Limited Stable:** superior migration, superior-medial erosion, extensive C-A arch acetabularization and femoralization of humeral head
- ◯ **Type 2B-Decentered, Unstable:** anterior-superior escape, absent stabilization, C-A arch and anterior structures deficient

* 95. Please grade the radiographs according to Hamada

- ◯ **Grade 1:** acromio-humeral interval (AHI) > 6mm
- ◯ **Grade 2:** acromio-humeral interval (AHI) 5mm or less
- ◯ **Grade 3:** grade 2 with acetabularization of acromion
- ◯ **Grade 4:** grade 3 with narrowing of gleno-humeral joint
- ◯ **Grade 5:** grade 4 with bony destruction and humeral head collapse

* 96. Please select a treatment

- ◯ non-operative (includes medications, injections, physical therapy, or watchful waiting)
- ◯ arthroscopic treatment
- ◯ hemiarthroplasty
- ◯ reverse shoulder arthroplasty

## Case 33

**An inactive 30 y.o. who is very low demand with their shoulder presents with moderate signs and symptoms (predominant symptoms that limit most things)**

* 97. Please grade the radiographs according to Seebauer

- ◯ **Type 1A-Centered, Stable:** minimal superior migration, C-A arch acetabularization and femoralization of humeral head
- ◯ **Type 1B-Centered, Medialized:** minimal superior migration, medial glenoid erosion, C-A arch acetabularization and femoralization of humeral head
- ◯ **Type 2A-Decentered, Limited Stable:** superior migration, superior-medial erosion, extensive C-A arch acetabularization and femoralization of humeral head
- ◯ **Type 2B-Decentered, Unstable:** anterior-superior escape, absent stabilization, C-A arch and anterior structures deficient

* 98. Please grade the radiographs according to Hamada

- ◯ **Grade 1:** acromio-humeral interval (AHI) > 6mm
- ◯ **Grade 2:** acromio-humeral interval (AHI) 5mm or less
- ◯ **Grade 3:** grade 2 with acetabularization of acromion
- ◯ **Grade 4:** grade 3 with narrowing of gleno-humeral joint
- ◯ **Grade 5:** grade 4 with bony destruction and humeral head collapse

* 99. Please select a treatment

- ◯ non-operative (includes medications, injections, physical therapy, or watchful waiting)
- ◯ arthroscopic treatment
- ◯ hemiarthroplasty
- ◯ reverse shoulder arthroplasty

## Case 34

**An active 30 y.o. who works as a manual laborer presents with severe signs and symptoms (constant)**

* 100. Please grade the radiographs according to Seebauer

- ◯ **Type 1A-Centered, Stable:** minimal superior migration, C-A arch acetabularization and femoralization of humeral head
- ◯ **Type 1B-Centered, Medialized:** minimal superior migration, medial glenoid erosion, C-A arch acetabularization and femoralization of humeral head
- ◯ **Type 2A-Decentered, Limited Stable:** superior migration, superior-medial erosion, extensive C-A arch acetabularization and femoralization of humeral head
- ◯ **Type 2B-Decentered, Unstable:** anterior-superior escape, absent stabilization, C-A arch and anterior structures deficient

* 101. Please grade the radiographs according to Hamada

- ◯ **Grade 1:** acromio-humeral interval (AHI) > 6mm
- ◯ **Grade 2:** acromio-humeral interval (AHI) 5mm or less
- ◯ **Grade 3:** grade 2 with acetabularization of acromion
- ◯ **Grade 4:** grade 3 with narrowing of gleno-humeral joint
- ◯ **Grade 5:** grade 4 with bony destruction and humeral head collapse

* 102. Please select a treatment

- ◯ non-operative (includes medications, injections, physical therapy, or watchful waiting)
- ◯ arthroscopic treatment
- ◯ hemiarthroplasty
- ◯ reverse shoulder arthroplasty

## Case 35

**A 30 y.o. who works a desk job and enjoys playing rec basketball and lifting weights presents with severe signs and symptoms (constant)**

* 103. Please grade the radiographs according to Seebauer

- ◯ **Type 1A-Centered, Stable:** minimal superior migration, C-A arch acetabularization and femoralization of humeral head
- ◯ **Type 1B-Centered, Medialized:** minimal superior migration, medial glenoid erosion, C-A arch acetabularization and femoralization of humeral head
- ◯ **Type 2A-Decentered, Limited Stable:** superior migration, superior-medial erosion, extensive C-A arch acetabularization and femoralization of humeral head
- ◯ **Type 2B-Decentered, Unstable:** anterior-superior escape, absent stabilization, C-A arch and anterior structures deficient

* 104. Please grade the radiographs according to Hamada

- ◯ **Grade 1:** acromio-humeral interval (AHI) > 6mm
- ◯ **Grade 2:** acromio-humeral interval (AHI) 5mm or less
- ◯ **Grade 3:** grade 2 with acetabularization of acromion
- ◯ **Grade 4:** grade 3 with narrowing of gleno-humeral joint
- ◯ **Grade 5:** grade 4 with bony destruction and humeral head collapse

* 105. Please select a treatment

- ◯ non-operative (includes medications, injections, physical therapy, or watchful waiting)
- ◯ arthroscopic treatment
- ◯ hemiarthroplasty
- ◯ reverse shoulder arthroplasty

## Case 36

**An inactive 30 y.o. who is very low demand with their shoulder presents with severe signs and symptoms (constant)**

* 106. Please grade the radiographs according to Seebauer

- **Type 1A-Centered, Stable:** minimal superior migration, C-A arch acetabularization and femoralization of humeral head
- **Type 1B-Centered, Medialized:** minimal superior migration, medial glenoid erosion, C-A arch acetabularization and femoralization of humeral head
- **Type 2A-Decentered, Limited Stable:** superior migration, superior-medial erosion, extensive C-A arch acetabularization and femoralization of humeral head
- **Type 2B-Decentered, Unstable:** anterior-superior escape, absent stabilization, C-A arch and anterior structures deficient

* 107. Please grade the radiographs according to Hamada

- **Grade 1:** acromio-humeral interval (AHI) > 6mm
- **Grade 2:** acromio-humeral interval (AHI) 5mm or less
- **Grade 3:** grade 2 with acetabularization of acromion
- **Grade 4:** grade 3 with narrowing of gleno-humeral joint
- **Grade 5:** grade 4 with bony destruction and humeral head collapse

* 108. Please select a treatment

- non-operative (includes medications, injections, physical therapy, or watchful waiting)
- arthroscopic treatment
- hemiarthroplasty
- reverse shoulder arthroplasty

## Case 37

**An active 45 y.o. who works as a manual laborer presents with mild signs and symptoms (only during strenuous activities)**

* 109. Please grade the radiographs according to Seebauer

- **Type 1A-Centered, Stable:** minimal superior migration, C-A arch acetabularization and femoralization of humeral head
- **Type 1B-Centered, Medialized:** minimal superior migration, medial glenoid erosion, C-A arch acetabularization and femoralization of humeral head
- **Type 2A-Decentered, Limited Stable:** superior migration, superior-medial erosion, extensive C-A arch acetabularization and femoralization of humeral head
- **Type 2B-Decentered, Unstable:** anterior-superior escape, absent stabilization, C-A arch and anterior structures deficient

* 110. Please grade the radiographs according to Hamada

- **Grade 1:** acromio-humeral interval (AHI) > 6mm
- **Grade 2:** acromio-humeral interval (AHI) 5mm or less
- **Grade 3:** grade 2 with acetabularization of acromion
- **Grade 4:** grade 3 with narrowing of gleno-humeral joint
- **Grade 5:** grade 4 with bony destruction and humeral head collapse

* 111. Please select a treatment

- non-operative (includes medications, injections, physical therapy, or watchful waiting)
- arthroscopic treatment
- hemiarthroplasty
- reverse shoulder arthroplasty

## Case 38

**A 45 y.o. who works a desk job and enjoys playing rec basketball and lifting weights presents with mild signs and symptoms (only during strenuous activities)**

* 112. Please grade the radiographs according to Seebauer

- **Type 1A-Centered, Stable:** minimal superior migration, C-A arch acetabularization and femoralization of humeral head
- **Type 1B-Centered, Medialized:** minimal superior migration, medial glenoid erosion, C-A arch acetabularization and femoralization of humeral head
- **Type 2A-Decentered, Limited Stable:** superior migration, superior-medial erosion, extensive C-A arch acetabularization and femoralization of humeral head
- **Type 2B-Decentered, Unstable:** anterior-superior escape, absent stabilization, C-A arch and anterior structures deficient

* 113. Please grade the radiographs according to Hamada

- **Grade 1:** acromio-humeral interval (AHI) > 6mm
- **Grade 2:** acromio-humeral interval (AHI) 5mm or less
- **Grade 3:** grade 2 with acetabularization of acromion
- **Grade 4:** grade 3 with narrowing of gleno-humeral joint
- **Grade 5:** grade 4 with bony destruction and humeral head collapse

* 114. Please select a treatment

- non-operative (includes medications, injections, physical therapy, or watchful waiting)
- arthroscopic treatment
- hemiarthroplasty
- reverse shoulder arthroplasty

## Case 39

**An inactive 45 y.o. who is very low demand with their shoulder presents with mild signs and symptoms (only during strenuous activities)**

* 115. Please grade the radiographs according to Seebauer

- ◯ **Type 1A-Centered, Stable:** minimal superior migration, C-A arch acetabularization and femoralization of humeral head
- ◯ **Type 1B-Centered, Medialized:** minimal superior migration, medial glenoid erosion, C-A arch acetabularization and femoralization of humeral head
- ◯ **Type 2A-Decentered, Limited Stable:** superior migration, superior-medial erosion, extensive C-A arch acetabularization and femoralization of humeral head
- ◯ **Type 2B-Decentered, Unstable:** anterior-superior escape, absent stabilization, C-A arch and anterior structures deficient

* 116. Please grade the radiographs according to Hamada

- ◯ **Grade 1:** acromio-humeral interval (AHI) > 6mm
- ◯ **Grade 2:** acromio-humeral interval (AHI) 5mm or less
- ◯ **Grade 3:** grade 2 with acetabularization of acromion
- ◯ **Grade 4:** grade 3 with narrowing of gleno-humeral joint
- ◯ **Grade 5:** grade 4 with bony destruction and humeral head collapse

* 117. Please select a treatment

- ◯ non-operative (includes medications, injections, physical therapy, or watchful waiting)
- ◯ arthroscopic treatment
- ◯ hemiarthroplasty
- ◯ reverse shoulder arthroplasty

## Case 40

**An active 45 y.o. who works as a manual laborer presents with moderate signs and symptoms (predominant symptoms that limit most things)**

* 118. Please grade the radiographs according to Seebauer

- ◯ **Type 1A-Centered, Stable:** minimal superior migration, C-A arch acetabularization and femoralization of humeral head
- ◯ **Type 1B-Centered, Medialized:** minimal superior migration, medial glenoid erosion, C-A arch acetabularization and femoralization of humeral head
- ◯ **Type 2A-Decentered, Limited Stable:** superior migration, superior-medial erosion, extensive C-A arch acetabularization and femoralization of humeral head
- ◯ **Type 2B-Decentered, Unstable:** anterior-superior escape, absent stabilization, C-A arch and anterior structures deficient

* 119. Please grade the radiographs according to Hamada

- ◯ **Grade 1:** acromio-humeral interval (AHI) > 6mm
- ◯ **Grade 2:** acromio-humeral interval (AHI) 5mm or less
- ◯ **Grade 3:** grade 2 with acetabularization of acromion
- ◯ **Grade 4:** grade 3 with narrowing of gleno-humeral joint
- ◯ **Grade 5:** grade 4 with bony destruction and humeral head collapse

* 120. Please select a treatment

- ◯ non-operative (includes medications, injections, physical therapy, or watchful waiting)
- ◯ arthroscopic treatment
- ◯ hemiarthroplasty
- ◯ reverse shoulder arthroplasty

## Case 41

**A 45 y.o. who works a desk job and enjoys playing rec basketball and lifting weights presents with moderate signs and symptoms (predominant symptoms that limit most things)**

* 121. Please grade the radiographs according to Seebauer

- ◯ **Type 1A-Centered, Stable:** minimal superior migration, C-A arch acetabularization and femoralization of humeral head
- ◯ **Type 1B-Centered, Medialized:** minimal superior migration, medial glenoid erosion, C-A arch acetabularization and femoralization of humeral head
- ◯ **Type 2A-Decentered, Limited Stable:** superior migration, superior-medial erosion, extensive C-A arch acetabularization and femoralization of humeral head
- ◯ **Type 2B-Decentered, Unstable:** anterior-superior escape, absent stabilization, C-A arch and anterior structures deficient

* 122. Please grade the radiographs according to Hamada

- ◯ **Grade 1:** acromio-humeral interval (AHI) > 6mm
- ◯ **Grade 2:** acromio-humeral interval (AHI) 5mm or less
- ◯ **Grade 3:** grade 2 with acetabularization of acromion
- ◯ **Grade 4:** grade 3 with narrowing of gleno-humeral joint
- ◯ **Grade 5:** grade 4 with bony destruction and humeral head collapse

* 123. Please select a treatment

- ◯ non-operative (includes medications, injections, physical therapy, or watchful waiting)
- ◯ arthroscopic treatment
- ◯ hemiarthroplasty
- ◯ reverse shoulder arthroplasty

## Case 42

**An inactive 45 y.o. who is very low demand with their shoulder presents with moderate signs and symptoms (predominant symptoms that limit most things)**

* 124. Please grade the radiographs according to Seebauer

- ◯ **Type 1A-Centered, Stable:** minimal superior migration, C-A arch acetabularization and femoralization of humeral head
- ◯ **Type 1B-Centered, Medialized:** minimal superior migration, medial glenoid erosion, C-A arch acetabularization and femoralization of humeral head
- ◯ **Type 2A-Decentered, Limited Stable:** superior migration, superior-medial erosion, extensive C-A arch acetabularization and femoralization of humeral head
- ◯ **Type 2B-Decentered, Unstable:** anterior-superior escape, absent stabilization, C-A arch and anterior structures deficient

* 125. Please grade the radiographs according to Hamada

- ◯ **Grade 1:** acromio-humeral interval (AHI) > 6mm
- ◯ **Grade 2:** acromio-humeral interval (AHI) 5mm or less
- ◯ **Grade 3:** grade 2 with acetabularization of acromion
- ◯ **Grade 4:** grade 3 with narrowing of gleno-humeral joint
- ◯ **Grade 5:** grade 4 with bony destruction and humeral head collapse

* 126. Please select a treatment

- ◯ non-operative (includes medications, injections, physical therapy, or watchful waiting)
- ◯ arthroscopic treatment
- ◯ hemiarthroplasty
- ◯ reverse shoulder arthroplasty

## Case 43

**An active 45 y.o. who works as a manual laborer presents with severe signs and symptoms (constant)**

* 127. Please grade the radiographs according to Seebauer

- **Type 1A-Centered, Stable:** minimal superior migration, C-A arch acetabularization and femoralization of humeral head
- **Type 1B-Centered, Medialized:** minimal superior migration, medial glenoid erosion, C-A arch acetabularization and femoralization of humeral head
- **Type 2A-Decentered, Limited Stable:** superior migration, superior-medial erosion, extensive C-A arch acetabularization and femoralization of humeral head
- **Type 2B-Decentered, Unstable:** anterior-superior escape, absent stabilization, C-A arch and anterior structures deficient

* 128. Please grade the radiographs according to Hamada

- **Grade 1:** acromio-humeral interval (AHI) > 6mm
- **Grade 2:** acromio-humeral interval (AHI) 5mm or less
- **Grade 3:** grade 2 with acetabularization of acromion
- **Grade 4:** grade 3 with narrowing of gleno-humeral joint
- **Grade 5:** grade 4 with bony destruction and humeral head collapse

* 129. Please select a treatment

- non-operative (includes medications, injections, physical therapy, or watchful waiting)
- arthroscopic treatment
- hemiarthroplasty
- reverse shoulder arthroplasty

## Case 44

**A 45 y.o. who works a desk job and enjoys playing rec basketball and lifting weights presents with severe signs and symptoms (constant)**

* 130. Please grade the radiographs according to Seebauer

- ◯ **Type 1A-Centered, Stable:** minimal superior migration, C-A arch acetabularization and femoralization of humeral head
- ◯ **Type 1B-Centered, Medialized:** minimal superior migration, medial glenoid erosion, C-A arch acetabularization and femoralization of humeral head
- ◯ **Type 2A-Decentered, Limited Stable:** superior migration, superior-medial erosion, extensive C-A arch acetabularization and femoralization of humeral head
- ◯ **Type 2B-Decentered, Unstable:** anterior-superior escape, absent stabilization, C-A arch and anterior structures deficient

* 131. Please grade the radiographs according to Hamada

- ◯ **Grade 1:** acromio-humeral interval (AHI) > 6mm
- ◯ **Grade 2:** acromio-humeral interval (AHI) 5mm or less
- ◯ **Grade 3:** grade 2 with acetabularization of acromion
- ◯ **Grade 4:** grade 3 with narrowing of gleno-humeral joint
- ◯ **Grade 5:** grade 4 with bony destruction and humeral head collapse

* 132. Please select a treatment

- ◯ non-operative (includes medications, injections, physical therapy, or watchful waiting)
- ◯ arthroscopic treatment
- ◯ hemiarthroplasty
- ◯ reverse shoulder arthroplasty

## Case 45

**An inactive 45 y.o. who is very low demand with their shoulder presents with severe signs and symptoms (constant)**

* 133. Please grade the radiographs according to Seebauer

- ◯ **Type 1A-Centered, Stable:** minimal superior migration, C-A arch acetabularization and femoralization of humeral head
- ◯ **Type 1B-Centered, Medialized:** minimal superior migration, medial glenoid erosion, C-A arch acetabularization and femoralization of humeral head
- ◯ **Type 2A-Decentered, Limited Stable:** superior migration, superior-medial erosion, extensive C-A arch acetabularization and femoralization of humeral head
- ◯ **Type 2B-Decentered, Unstable:** anterior-superior escape, absent stabilization, C-A arch and anterior structures deficient

* 134. Please grade the radiographs according to Hamada

- ◯ **Grade 1:** acromio-humeral interval (AHI) > 6mm
- ◯ **Grade 2:** acromio-humeral interval (AHI) 5mm or less
- ◯ **Grade 3:** grade 2 with acetabularization of acromion
- ◯ **Grade 4:** grade 3 with narrowing of gleno-humeral joint
- ◯ **Grade 5:** grade 4 with bony destruction and humeral head collapse

* 135. Please select a treatment

- ◯ non-operative (includes medications, injections, physical therapy, or watchful waiting)
- ◯ arthroscopic treatment
- ◯ hemiarthroplasty
- ◯ reverse shoulder arthroplasty

## Case 46

**An active 65 y.o. who works as a manual laborer presents with mild signs and symptoms (only during strenuous activities)**

* 136. Please grade the radiographs according to Seebauer

- **Type 1A-Centered, Stable:** minimal superior migration, C-A arch acetabularization and femoralization of humeral head
- **Type 1B-Centered, Medialized:** minimal superior migration, medial glenoid erosion, C-A arch acetabularization and femoralization of humeral head
- **Type 2A-Decentered, Limited Stable:** superior migration, superior-medial erosion, extensive C-A arch acetabularization and femoralization of humeral head
- **Type 2B-Decentered, Unstable:** anterior-superior escape, absent stabilization, C-A arch and anterior structures deficient

* 137. Please grade the radiographs according to Hamada

- **Grade 1:** acromio-humeral interval (AHI) > 6mm
- **Grade 2:** acromio-humeral interval (AHI) 5mm or less
- **Grade 3:** grade 2 with acetabularization of acromion
- **Grade 4:** grade 3 with narrowing of gleno-humeral joint
- **Grade 5:** grade 4 with bony destruction and humeral head collapse

* 138. Please select a treatment

- non-operative (includes medications, injections, physical therapy, or watchful waiting)
- arthroscopic treatment
- hemiarthroplasty
- reverse shoulder arthroplasty

## Case 47

**A 65 y.o. who works a desk job and enjoys playing rec basketball and lifting weights presents with mild signs and symptoms (only during strenuous activities)**

* 139. Please grade the radiographs according to Seebauer

- ○ **Type 1A-Centered, Stable:** minimal superior migration, C-A arch acetabularization and femoralization of humeral head
- ○ **Type 1B-Centered, Medialized:** minimal superior migration, medial glenoid erosion, C-A arch acetabularization and femoralization of humeral head
- ○ **Type 2A-Decentered, Limited Stable:** superior migration, superior-medial erosion, extensive C-A arch acetabularization and femoralization of humeral head
- ○ **Type 2B-Decentered, Unstable:** anterior-superior escape, absent stabilization, C-A arch and anterior structures deficient

* 140. Please grade the radiographs according to Hamada

- ○ **Grade 1:** acromio-humeral interval (AHI) > 6mm
- ○ **Grade 2:** acromio-humeral interval (AHI) 5mm or less
- ○ **Grade 3:** grade 2 with acetabularization of acromion
- ○ **Grade 4:** grade 3 with narrowing of gleno-humeral joint
- ○ **Grade 5:** grade 4 with bony destruction and humeral head collapse

* 141. Please select a treatment

- ○ non-operative (includes medications, injections, physical therapy, or watchful waiting)
- ○ arthroscopic treatment
- ○ hemiarthroplasty
- ○ reverse shoulder arthroplasty

## Case 48

**An inactive 65 y.o. who is very low demand with their shoulder presents with mild signs and symptoms (only during strenuous activities)**

* 142. Please grade the radiographs according to Seebauer

- ◯ **Type 1A-Centered, Stable:** minimal superior migration, C-A arch acetabularization and femoralization of humeral head
- ◯ **Type 1B-Centered, Medialized:** minimal superior migration, medial glenoid erosion, C-A arch acetabularization and femoralization of humeral head
- ◯ **Type 2A-Decentered, Limited Stable:** superior migration, superior-medial erosion, extensive C-A arch acetabularization and femoralization of humeral head
- ◯ **Type 2B-Decentered, Unstable:** anterior-superior escape, absent stabilization, C-A arch and anterior structures deficient

* 143. Please grade the radiographs according to Hamada

- ◯ **Grade 1:** acromio-humeral interval (AHI) > 6mm
- ◯ **Grade 2:** acromio-humeral interval (AHI) 5mm or less
- ◯ **Grade 3:** grade 2 with acetabularization of acromion
- ◯ **Grade 4:** grade 3 with narrowing of gleno-humeral joint
- ◯ **Grade 5:** grade 4 with bony destruction and humeral head collapse

* 144. Please select a treatment

- ◯ non-operative (includes medications, injections, physical therapy, or watchful waiting)
- ◯ arthroscopic treatment
- ◯ hemiarthroplasty
- ◯ reverse shoulder arthroplasty

## Case 49

**An active 65 y.o. who works as a manual laborer presents with moderate signs and symptoms (predominant symptoms that limit most things)**

* 145. Please grade the radiographs according to Seebauer

- ◯ **Type 1A-Centered, Stable:** minimal superior migration, C-A arch acetabularization and femoralization of humeral head
- ◯ **Type 1B-Centered, Medialized:** minimal superior migration, medial glenoid erosion, C-A arch acetabularization and femoralization of humeral head
- ◯ **Type 2A-Decentered, Limited Stable:** superior migration, superior-medial erosion, extensive C-A arch acetabularization and femoralization of humeral head
- ◯ **Type 2B-Decentered, Unstable:** anterior-superior escape, absent stabilization, C-A arch and anterior structures deficient

* 146. Please grade the radiographs according to Hamada

- ◯ **Grade 1:** acromio-humeral interval (AHI) > 6mm
- ◯ **Grade 2:** acromio-humeral interval (AHI) 5mm or less
- ◯ **Grade 3:** grade 2 with acetabularization of acromion
- ◯ **Grade 4:** grade 3 with narrowing of gleno-humeral joint
- ◯ **Grade 5:** grade 4 with bony destruction and humeral head collapse

* 147. Please select a treatment

- ◯ non-operative (includes medications, injections, physical therapy, or watchful waiting)
- ◯ arthroscopic treatment
- ◯ hemiarthroplasty
- ◯ reverse shoulder arthroplasty

## Case 50

**A 65 y.o. who works a desk job and enjoys playing rec basketball and lifting weights presents with moderate signs and symptoms (predominant symptoms that limit most things)**

* 148. Please grade the radiographs according to Seebauer

- ◯ **Type 1A-Centered, Stable:** minimal superior migration, C-A arch acetabularization and femoralization of humeral head
- ◯ **Type 1B-Centered, Medialized:** minimal superior migration, medial glenoid erosion, C-A arch acetabularization and femoralization of humeral head
- ◯ **Type 2A-Decentered, Limited Stable:** superior migration, superior-medial erosion, extensive C-A arch acetabularization and femoralization of humeral head
- ◯ **Type 2B-Decentered, Unstable:** anterior-superior escape, absent stabilization, C-A arch and anterior structures deficient

* 149. Please grade the radiographs according to Hamada

- ◯ **Grade 1:** acromio-humeral interval (AHI) > 6mm
- ◯ **Grade 2:** acromio-humeral interval (AHI) 5mm or less
- ◯ **Grade 3:** grade 2 with acetabularization of acromion
- ◯ **Grade 4:** grade 3 with narrowing of gleno-humeral joint
- ◯ **Grade 5:** grade 4 with bony destruction and humeral head collapse

* 150. Please select a treatment

- ◯ non-operative (includes medications, injections, physical therapy, or watchful waiting)
- ◯ arthroscopic treatment
- ◯ hemiarthroplasty
- ◯ reverse shoulder arthroplasty

## Case 51

**An inactive 65 y.o. who is very low demand with their shoulder presents with moderate signs and symptoms (predominant symptoms that limit most things)**

* 151. Please grade the radiographs according to Seebauer

- **Type 1A-Centered, Stable:** minimal superior migration, C-A arch acetabularization and femoralization of humeral head
- **Type 1B-Centered, Medialized:** minimal superior migration, medial glenoid erosion, C-A arch acetabularization and femoralization of humeral head
- **Type 2A-Decentered, Limited Stable:** superior migration, superior-medial erosion, extensive C-A arch acetabularization and femoralization of humeral head
- **Type 2B-Decentered, Unstable:** anterior-superior escape, absent stabilization, C-A arch and anterior structures deficient

* 152. Please grade the radiographs according to Hamada

- **Grade 1:** acromio-humeral interval (AHI) > 6mm
- **Grade 2:** acromio-humeral interval (AHI) 5mm or less
- **Grade 3:** grade 2 with acetabularization of acromion
- **Grade 4:** grade 3 with narrowing of gleno-humeral joint
- **Grade 5:** grade 4 with bony destruction and humeral head collapse

* 153. Please select a treatment

- non-operative (includes medications, injections, physical therapy, or watchful waiting)
- arthroscopic treatment
- hemiarthroplasty
- reverse shoulder arthroplasty

## Case 52

**An active 65 y.o. who works as a manual laborer presents with severe signs and symptoms (constant)**

* 154. Please grade the radiographs according to Seebauer

- ○ **Type 1A-Centered, Stable:** minimal superior migration, C-A arch acetabularization and femoralization of humeral head
- ○ **Type 1B-Centered, Medialized:** minimal superior migration, medial glenoid erosion, C-A arch acetabularization and femoralization of humeral head
- ○ **Type 2A-Decentered, Limited Stable:** superior migration, superior-medial erosion, extensive C-A arch acetabularization and femoralization of humeral head
- ○ **Type 2B-Decentered, Unstable:** anterior-superior escape, absent stabilization, C-A arch and anterior structures deficient

* 155. Please grade the radiographs according to Hamada

- ○ **Grade 1:** acromio-humeral interval (AHI) > 6mm
- ○ **Grade 2:** acromio-humeral interval (AHI) 5mm or less
- ○ **Grade 3:** grade 2 with acetabularization of acromion
- ○ **Grade 4:** grade 3 with narrowing of gleno-humeral joint
- ○ **Grade 5:** grade 4 with bony destruction and humeral head collapse

* 156. Please select a treatment

- ○ non-operative (includes medications, injections, physical therapy, or watchful waiting)
- ○ arthroscopic treatment
- ○ hemiarthroplasty
- ○ reverse shoulder arthroplasty

## Case 53

**A 65 y.o. who works a desk job and enjoys playing rec basketball and lifting weights presents with severe signs and symptoms (constant)**

* 157. Please grade the radiographs according to Seebauer

- ◯ **Type 1A-Centered, Stable:** minimal superior migration, C-A arch acetabularization and femoralization of humeral head
- ◯ **Type 1B-Centered, Medialized:** minimal superior migration, medial glenoid erosion, C-A arch acetabularization and femoralization of humeral head
- ◯ **Type 2A-Decentered, Limited Stable:** superior migration, superior-medial erosion, extensive C-A arch acetabularization and femoralization of humeral head
- ◯ **Type 2B-Decentered, Unstable:** anterior-superior escape, absent stabilization, C-A arch and anterior structures deficient

* 158. Please grade the radiographs according to Hamada

- ◯ **Grade 1:** acromio-humeral interval (AHI) > 6mm
- ◯ **Grade 2:** acromio-humeral interval (AHI) 5mm or less
- ◯ **Grade 3:** grade 2 with acetabularization of acromion
- ◯ **Grade 4:** grade 3 with narrowing of gleno-humeral joint
- ◯ **Grade 5:** grade 4 with bony destruction and humeral head collapse

* 159. Please select a treatment

- ◯ non-operative (includes medications, injections, physical therapy, or watchful waiting)
- ◯ arthroscopic treatment
- ◯ hemiarthroplasty
- ◯ reverse shoulder arthroplasty

## Case 54

**An inactive 65 y.o. who is very low demand with their shoulder presents with severe signs and symptoms (constant)**

* 160. Please grade the radiographs according to Seebauer

- ◯ **Type 1A-Centered, Stable:** minimal superior migration, C-A arch acetabularization and femoralization of humeral head
- ◯ **Type 1B-Centered, Medialized:** minimal superior migration, medial glenoid erosion, C-A arch acetabularization and femoralization of humeral head
- ◯ **Type 2A-Decentered, Limited Stable:** superior migration, superior-medial erosion, extensive C-A arch acetabularization and femoralization of humeral head
- ◯ **Type 2B-Decentered, Unstable:** anterior-superior escape, absent stabilization, C-A arch and anterior structures deficient

* 161. Please grade the radiographs according to Hamada

- ◯ **Grade 1:** acromio-humeral interval (AHI) > 6mm
- ◯ **Grade 2:** acromio-humeral interval (AHI) 5mm or less
- ◯ **Grade 3:** grade 2 with acetabularization of acromion
- ◯ **Grade 4:** grade 3 with narrowing of gleno-humeral joint
- ◯ **Grade 5:** grade 4 with bony destruction and humeral head collapse

* 162. Please select a treatment

- ◯ non-operative (includes medications, injections, physical therapy, or watchful waiting)
- ◯ arthroscopic treatment
- ◯ hemiarthroplasty
- ◯ reverse shoulder arthroplasty
